# Effect of omega-3 fatty acids on cardiovascular outcomes: A systematic review and meta-analysis

**DOI:** 10.1016/j.eclinm.2021.100997

**Published:** 2021-07-08

**Authors:** Safi U. Khan, Ahmad N. Lone, Muhammad Shahzeb Khan, Salim S. Virani, Roger S. Blumenthal, Khurram Nasir, Michael Miller, Erin D. Michos, Christie M. Ballantyne, William E. Boden, Deepak L. Bhatt

**Affiliations:** a Department of Medicine, West Virginia University, Morgantown, WV, United States; b Department of Medicine, University of Mississippi Medical Center, Jackson, MS, United States; c Michael E. DeBakey Veterans Affair Medical Center & Department of Medicine, Baylor College of Medicine, Houston, TX, United States; d Ciccarone Center for the Prevention of Cardiovascular Disease, Johns Hopkins School of Medicine, Baltimore, MD, United States; e Division of Cardiology, Johns Hopkins School of Medicine, Baltimore, MD, United States; f Outcomes Research, Houston Methodist, Houston, TX, United States; g Division of Cardiovascular Prevention and Wellness, Department of Cardiology, Houston Methodist DeBakey Heart & Vascular Center, Houston, TX, United States; h Department of Medicine, Division of Cardiology, University of Maryland Medical Center, Baltimore, MD, United States; i VA New England Healthcare System, Boston University School of Medicine, Boston, MA, United States; j Brigham and Women's Hospital Heart and Vascular Center, Harvard Medical School, 75 Francis Street, Boston, MA 02115, United States

**Keywords:** Omega-3 fatty acid, Eicosapentaenoic acid, Docosahexaenoic acid, Meta-analysis

## Abstract

**Background:**

The effects of omega-3 fatty acids (FAs), such as eicosapentaenoic (EPA) and docosahexaenoic (DHA) acids, on cardiovascular outcomes are uncertain. We aimed to determine the effectiveness of omega-3 FAs on fatal and non-fatal cardiovascular outcomes and examine the potential variability in EPA vs. EPA+DHA treatment effects.

**Methods:**

We searched EMBASE, PubMed, ClinicalTrials.gov, and Cochrane library databases through June 7, 2021. We performed a meta-analysis of 38 randomized controlled trials of omega-3 FAs, stratified by EPA monotherapy and EPA+DHA therapy. We estimated random-effects rate ratios (RRs) with (95% confidence intervals) and rated the certainty of evidence using GRADE. The key outcomes of interest were cardiovascular mortality, non-fatal cardiovascular outcomes, bleeding, and atrial fibrillation (AF). The protocol was registered in PROSPERO (CRD42021227580).

**Findings:**

In 149,051 participants, omega-3 FA was associated with reducing cardiovascular mortality (RR, 0.93 [0.88-0.98]; *p* = 0.01), non-fatal myocardial infarction (MI) (RR, 0.87 [0.81–0.93]; *p* = 0.0001), coronary heart disease events (CHD) (RR, 0.91 [0.87–0.96]; *p* = 0.0002), major adverse cardiovascular events (MACE) (RR, 0.95 [0.92–0.98]; *p* = 0.002), and revascularization (RR, 0.91 [0.87–0.95]; *p* = 0.0001). The meta-analysis showed higher RR reductions with EPA monotherapy (0.82 [0.68–0.99]) than with EPA + DHA (0.94 [0.89–0.99]) for cardiovascular mortality, non-fatal MI (EPA: 0.72 [0.62–0.84]; EPA+DHA: 0.92 [0.85–1.00]), CHD events (EPA: 0.73 [0.62–0.85]; EPA+DHA: 0.94 [0.89–0.99]), as well for MACE and revascularization. Omega-3 FA increased incident AF (RR, 1.26 [1.08–1.48]). EPA monotherapy vs. control was associated with a higher risk of total bleeding (RR: 1.49 [1.20–1.84]) and AF (RR, 1.35 [1.10–1.66]).

**Interpretation:**

Omega-3 FAs reduced cardiovascular mortality and improved cardiovascular outcomes. The cardiovascular risk reduction was more prominent with EPA monotherapy than with EPA+DHA.

**Funding:**

None.

**Research in context**

**Evidence before this study**

Eicosapentaenoic acid (EPA) and docosahexaenoic acid (DHA) differ in their biological effects on membrane structure and lipid metabolism. Therefore, combining DHA with the EPA may modify the clinical effects of EPA treatment. Previous meta-analyses have combined the EPA with EPA+DHA trials, which might have masked the effects of individual formulations of omega-3 fatty acids (FAs).

**Added value of this study**

In this updated meta-analysis of 38 randomized controlled trials, omega-3 FAs were associated with reducing cardiovascular mortality and other cardiovascular outcomes. A meta-analysis of EPA trials showed greater relative risk reductions in cardiovascular outcomes than those of EPA+DHA.

**Implications of all the available evidence**

Several clinical guidelines recommendations endorsed purified ethyl ester of EPA after REDUCE-IT. However, two recent negative trials of EPA + DHA, STRENGTH and OMEMI, have put under discussion the utility of omega-3 FAs in preventing atherosclerotic cardiovascular events. This study provides evidence regarding the therapeutic efficacy of omega-3 FAs and may explain the conflicting results between EPA monotherapy trials and those with EPA+DHA.

Alt-text: Unlabelled box

## 1 Introduction

Omega-3 fatty acids (FAs), such as eicosapentaenoic (EPA) and docosahexaenoic (DHA) acids, may reduce the risk of atherosclerotic cardiovascular disease (ASCVD) events through various mechanisms, including triglyceride (TG) lowering, membrane stabilization, and antithrombotic, anti-inflammatory, or antiarrhythmic properties [1]. Consequently, randomized controlled trials explored the cardiovascular effects of omega-3 FAs with considerable interest. The JELIS (the JAPAN EPA Lipid Intervention Study) trial [2] showed a reduction in major coronary events in patients with hypercholesteremia using purified EPA. However, the study was limited by open-label design, lack of placebo group, the inclusion of potentially subjective endpoints in the primary composite outcome (e.g., unstable angina and elective revascularization), and lower intensity of statin therapy in participants with average low-density lipoprotein cholesterol (LDL-C) of ~180 mg/dL at baseline.

In 2018, three randomized controlled trials examining different preparations of omega-3 FAs showed divergent results [[3], [4], [5]]. The ASCEND (A Study of Cardiovascular Events in Diabetes) [3] and VITAL (Vitamin D and Omega-3 Trial) trials [5] using EPA+DHA did not significantly reduce the primary cardiovascular endpoints (ASCEND: non-fatal myocardial infarction [MI] or ischemic stroke, transient ischemic attack, or vascular death excluding confirmed intracranial hemorrhage [3]; VITAL: MI, stroke, or death from cardiovascular causes) [5]. Conversely, REDUCE-IT (Reduction of Cardiovascular Events with Icosapent Ethyl-Intervention Trial) [4] showed a significant 25% relative reduction in the primary composite efficacy endpoint of cardiovascular death, non-fatal MI, non-fatal stroke, coronary revascularization, or unstable angina (an absolute reduction of 4.8%) with icosapent ethyl — a highly purified ethyl ester of EPA — in patients with established ASCVD or those with high risk for ASCVD (diabetes with at least one additional risk factor). The key secondary endpoint of cardiovascular death, MI, or stroke was significantly reduced by 26%, and death from cardiovascular causes was significantly reduced by 20%.

Two studies in 2020, STRENGTH (the Long-Term Outcomes Study to Assess Statin Residual Risk with Epanova in High Cardiovascular Risk Patients with Hypertriglyceridemia) [6] and OMEMI (the Omega-3 fatty acids in Elderly with Myocardial Infarction) [7], showed null results for combined EPA+DHA therapy on the primary endpoints (STRENGTH: composite of cardiovascular death, non-fatal MI, nonfatal stroke, emergent/elective coronary revascularization, or unstable angina requiring hospitalization [6], OMEMI: non-fatal MI, unscheduled revascularization, stroke or all-cause mortality) [7]. These discordant trial results have led to considerable uncertainty and debate about the potential role of omega-3 FAs in reducing ASCVD residual risk. Moreover, since EPA and DHA differ in their biological effects on membrane structure and lipid metabolism, this variability led to the hypothesis that combining DHA with the EPA might partially offset the beneficial clinical effects of EPA treatment alone [1,8]. To explore this potential clinical heterogeneity across omega-3 FAs trials, we performed an updated systematic review and meta-analysis with a primary focus on determining the effectiveness and safety of omega-3 FAs on fatal and non-fatal cardiovascular outcomes in adults. The secondary focus was on examining the potential variability in the effects generated by EPA vs. EPA+DHA treatment.

## 2 Methods

We followed the PRISMA (Preferred Reporting Items for Systematic Reviews and Meta-Analysis) and the Cochrane Collaboration guidelines for this meta-analysis [9,10]. The protocol was registered in https://www.crd.york.ac.uk/PROSPERO/; Unique identifier: CRD42021227580.

### 2.1 Search strategy and selection criteria

We performed a comprehensive literature search, without language restriction, using the electronic databases of EMBASE, PubMed, ClinicalTrials.gov, and Cochrane library, through June 7, 2021. We also searched Web sites of major cardiovascular and medicine journals (www.nejm.org; https://www.thelancet.com/; https://jamanetwork.com; http://annals.org/aim; https://academic.oup.com/eurheartj; www.onlinejacc.org; and ww.ahajournals.org/journal/circ), and bibliographies of relevant studies [[11], [12], [13]]. Our broad search strategy included a combination of the following general search terms: “omega-3 fatty acid,” “eicosapentaenoic acid,” “docosahexaenoic acid,” “fish oil,” “cholesterol,” “triglycerides,” “cardiovascular disease,” etc. (*Appendix p2*).

The pre-determined inclusion criteria were (1) randomized controlled trials that compared omega-3 FA intake (EPA or EPA+DHA) vs. control (placebo, no supplementation, or lower dose of omega-3 FA) in adults; (2) follow-up duration of at least 12 months; and (3) trials must report mortality and cardiovascular outcomes of interest. Consistent with the prior report [12], we excluded trials where intervention consisted of dietary advice, owing to implicit variability in the amounts of EPA and DHA reported in food items and therefore unreliable readouts of the effect of omega-3 FA acid intake on clinical outcomes. There were no limitations on language or sample size.

We removed the duplicates, and following the study selection criteria, we screened the remaining articles at the title and abstract level and then at the full-text level. The process of study search and selection was performed independently by investigators (S.U.K. and A.N.L). Any conflicts were resolved by discussion, mutual consensus, and referring to the original study.

### 2.2 Data extraction

Two authors (S.U.K. and A.N.L) independently abstracted and adjudicated data on pre-specified data collection forms and resolved any discrepancies by consensus. We outlined data on characteristics of the trials and participants (age, sex, comorbidities, treatment arms and their composition [EPA vs. EPA+DHA with dosages]), control arms (active vs. placebo), follow-up duration, crude point estimates, events, and sample sizes for desired endpoints. Our efficacy outcomes of interest were cardiovascular mortality, all-cause mortality, non-fatal MI, coronary heart disease (CHD) events, major adverse cardiovascular events (MACE), revascularization, non-fatal stroke, ischemic stroke, and hemorrhagic stroke. The safety endpoints included atrial fibrillation (AF), total bleeding, major and minor bleeding, and gastrointestinal adverse events. We defined CHD events as the number of participants experiencing the first occurrence of CHD or coronary events, total MI, acute coronary syndrome, or stable or unstable angina [13]. We abstracted data on the intention-to-treat principle.

### 2.3 Risk of bias within individual studies

We used Cochrane criteria for assessing the risk of bias for each trial across the following domains: randomization, allocation concealment, blinding of participants and personnel, blinding of outcome assessors, selective outcome reporting, incomplete outcome data, and others. We rated trials as (i) low risk of bias, (ii) some concerns — probably low risk of bias, (iii) some concerns — probably high risk of bias, or (iv) high risk of bias. Trials were rated as high risk of bias overall if one or more domains were rated probably high risk of bias or high risk of bias, and as low risk of bias if all domains were rated probably low risk of bias or low risk of bias. Two investigators (S.U.K and A.N.L) independently appraised the potential risks of bias and resolved the discrepancies by discussion (*Appendix p 3 and 4*).

### 2.4 Data synthesis and summary measures

We performed a pairwise meta-analysis using a frequentist framework for all patients regardless of dosages, baseline therapy, and setting (primary vs. secondary prevention). We reported effect sizes as risk differences (RDs) and rate ratios (RRs) with 95% confidence intervals (CI). We derived RRs and RDs from an analysis with adjusted models by person-years to account for potential differences in follow-up duration across trials. We calculated RDs from RRs and the baseline risk of the population [14]. We used MACE risk in the control group as a proxy for population-level baseline risk. RDs were reported as incident cases per 1000 person-years.

### 2.5 Statistical analysis

We pooled outcomes using a random-effects model. The DerSimonian and Laird method was used for the estimation of τ [15]. We used *I*^2^ statistics to measure the extent of unexplained statistical heterogeneity: *I*^2^ greater than 50% was considered a high degree of between-study statistical heterogeneity [16]. We stratified the analyses by *EPA* and *EPA+DHA* to compare the effects of both components of omega-3 fatty acids on outcomes. Publication bias was assessed using a funnel plot and Egger's regression test.

To explore potential sources of heterogeneity, additional subgroup analyses were performed according to age, type of control (active vs. placebo), population (primary or secondary prevention), risk of cardiovascular disease events (low/moderate, high; *Appendix p 5 and 6)*, and risk of bias (low and high). Sensitivity analyses comprised the fixed-effects model, leave-one-out meta-analysis, exclusion of trials with a high risk of bias, and exclusion of trials with low/moderate cardiovascular disease risk. For all analyses, statistical significance was set at 5%. Comprehensive meta-analysis V 3.0 (Biostat, Englewood, NJ, USA) was used.

### 2.6 The certainty of the evidence

We evaluated the certainty of evidence using the grading of recommendations assessment, development, and evaluation (GRADE) approach (https://gdt.gradepro.org/app [17]. Two experienced authors (S.U.K. and A.N.L.) rated each domain separately for each comparison, and any discrepancies were resolved by consensus. The certainty for each comparison and outcome was rated as high, moderate, low, or very low, based on considerations of risk of bias, inconsistency, imprecision, indirectness, and other considerations (publication bias, large effect, plausible confounding, and dose-response gradient). The summary of the GRADE evidence chart is reported on *Appendix p 7 and 8.*

### 2.7 Role of the funding source

This study received no specific grant from any funding agency in the public, commercial, or not-for-profit sectors. S. U. Khan and A. N. Lone accessed the data. S. U. Khan and D. L. Bhatt decided to submit for publication. All the co-authors agreed with the decision.

## 3 Results

**Fig. 1:**
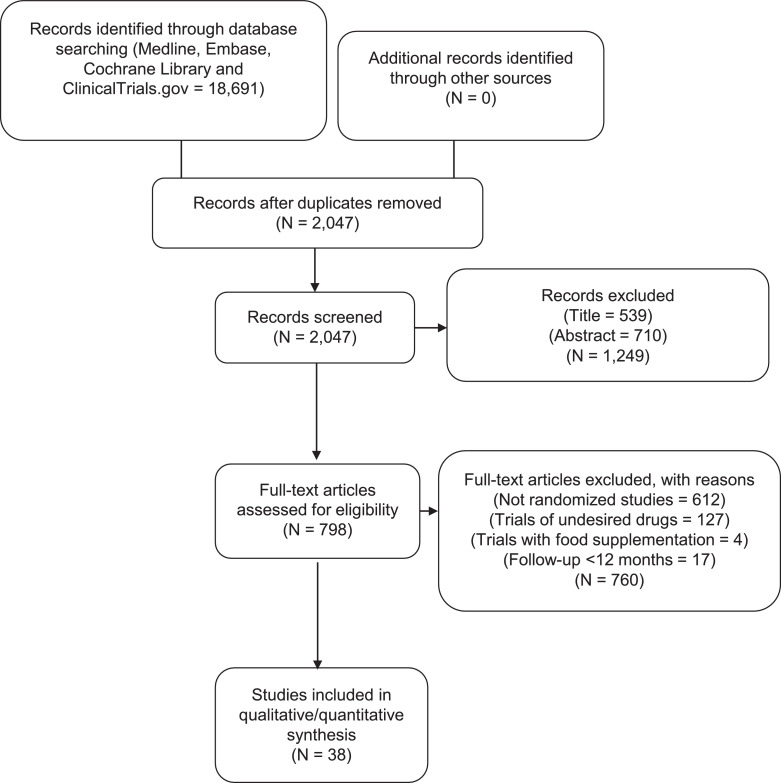
Flow chart showing study selection process.

We reviewed a total of 798 articles for eligibility after removing duplicates and screening at the title and abstract level. Further, 760 articles were removed based on a priori study selection criteria. Ultimately, 38 trials encompassing 149,051 patients were included (Fig. 1). The characteristics of the participants and trials are reported in Table 1. Of 38 trials, 4 trials compared EPA vs. control and 34 trials compared EPA+DHA vs. control. Twenty-two trials studied primary prevention. The dose of omega-3 FAs ranged from 0.4 g/day to 5.5 g/day. The EPA trials had dose ranges from 1.8 to 4.0 g/day and EPA+DHA from 0.4 to 5.5 g/day. The patients’ mean age ranged from 39 to 78 years, and the proportion of enrolled women varied from 0% to 77.5%. The median follow-up duration across the trials was 2.0 years (IQR, 1–4.2).

**Table 1:** Baseline characteristics of trials and participants.

		No. (%)							Baseline levels, mg/dL				Baseline levels, mg/dL			
Trial/Author, *y*	Age, *y*	Women	Coronary artery disease	Hypertension	Diabetes	Treatment	Patients	Dose, g/day	TG	LDL-C	Control	Patients	TG	LDL-C	StatinNo. (%)	Follow-up, *y*
Alpha Omega, 2010[35]	69	504 (20.8)	2,428 (100)	NR	511 (21)	EPA + DHA	2404	0.4	144.3	101.5	Alpha linoleic acid	2433	149.6	100.4	2,064 (85)	3.3
AFFORD, 2013[36]	61	105 (31.6)	43 (13.6)	146 (43.5)	26 (8.2)	EPA + DHA	165	2.40	—	—	Safflower oil	172	—	—	—	1.0
AREDS2, 2014[37]	74.3	2,387 (56.8)	405 (9.5)	—	546 (13)	EPA + DHA	2147	1.0	—	—	*Supplements	2056	—	—	1,849 (44)	5.0
ASCEND, 2018[3]	63.3	5,796 (37.4)	0	9536 (62.6)	15,480 (100)	EPA + DHA	7740	0.84	—	113	Olive oil	7740	—	112	11,653 (75.3)	7.4
Brox et al., 2001[38]	55	60 (50)	0	—	0	EPA + DHA	80	3.0	—	—	No treatment	40	—	—	0	1.2
Baldassarre et al., 2006[39]	53.7	6 (9.4)	0	0	0	EPA + DHA	32	1.8	276.1	154.4	Olive oil	32	270.8	150.2	0	2.0
DO IT, 2010[40]	70	0	0	157 (28)	82 (14.5)	EPA + DHA	282	1.32	150.4	158.3	Corn oil	281	150.4	154.4	—	3.0
Derosa et al., 2016[41]	54.1	141 (50.1)	—	—	0	EPA + DHA	138	2.55	182.5	127.5	No treatment	143	188.2	124.1	—	1.5
EPIC-1, 2008[42]	39.4	201 (55.4)	—	—	—	EPA + DHA	188	3.0	—	—	Medium chain triglycerides	186	—	—	—	4.3
EPE-A, 2014[43]	48.7	148 (60.9)	0	—	85 (35)	EPA	168	2.7	149	111	No treatment	75	139	120	0	1.0
ENRGISE, 2018[44]	77.6	137 (47.4)	—	200 (69.2)	68 (23.5)	EPA + DHA	148	1.8	—	—	Corn oil	141	—	—	—	1.0
FAAT, 2005[45]	65.5	68 (16.9)	314 (78.1)	—	—	EPA + DHA	200	2.6	—	—	Olive oil	202	—	—	—	1.0
FORWARD, 2013[46]	66.1	265 (45.2)	67 (11.7)	536 (91.5)	74 (12.9)	EPA + DHA	289	0.85	—	—	Olive oil	297	—	—	—	1.0
FOSTAR, 2016[47]	61	100 (50)	—	—	—	EPA + DHA	101	4.5	—	—	Sunola oil	101	—	—	—	2.0
GISSI-P, 1999[48]	59.4	854 (15.1)	5,664 (100)	4025 (35.5)	831 (14.6)	EPA + DHA	5666	0.87	162.6	137.3	No treatment	5658	16—1.9	138.5	—	3.5
GISSI-HF, 2008[19]	67	1,516 (21.7)	3,467 (49.7)	3808 (54.6)	1,974 (28.3)	EPA + DHA	3494	0.87	—	—	Olive oil	3481	—	—	1,579 (22.7)	3.9
HARP, 1995[49]	62	5 (6.5)	80 (100)	34 (42.2)	8 (13.6)	EPA + DHA	41	0.4	128	122	Olive oil	39	137	117	—	2.0
HEARTS, 2017[50]	63.0	208 (85)	240 (100)	206 (84)	68 (28.3)	EPA + DHA	143	3.36	123.0	78.5	No treatment	142	117.0	77.5	245 (100)	2.5
JELIS, 2007[2]	61	12,786 (68.6)	—	6619 (35.5)	3,040 (16)	EPA	9326	1.8	153.1	181.1	No treatment	9319	154.0	181.5	18,003 (96.6)	4.6
Kumar et al., 2012[51]	62	141 (77.5)	31 (17.4)	95 (51.2)	27 (15.2)	EPA + DHA	92	1.7	—	—	No treatment	90	—	—	68 (38.2)	1.0
MAPT, 2017[52]	75.3	978 (64)	—	—	—	EPA + DHA	840	1.03	—	—	Paraffin oil	840	—	—	—	3.0
Nye, 1990[53]	54	17 (23)	73 (100)	—	—	EPA + DHA	36	3.6	—	—	Olive oil	37	—	—	—	1.0
Nosaka et al., 2017[54]	70.5	56 (23.5)	238 (100)	167 (70)	92 (38.7)	EPA	119	1.8	117	118	No treatment	119	105	116	238 (100)	1.0
OFAMI, 2001[55]	64	62 (26.1)	238 (100)	78 (26)	31 (10.4)	EPA + DHA	150	3.36	145.1	—	Corn oil	150	137.2	—	107 (45.0)	1.5
ORIGIN, 2012[56]	63.5	4,386 (35)	—	9962 (79.5)	—	EPA + DHA	6281	0.84	142	112	Olive oil	6255	140	112	6,739 (53.8)	6.2
OMEGA, 2009[57]	64	977 (25.6)	3,818 (100)	2561 (66.5)	1,032 (27)	EPA + DHA	1940	0.85	—	—	Olive oil	1911	—	—	3,123 (81.5)	1.0
OMEMI, 2020[7]	74	294 (29)	1,014 (100)	611 (60.3)	210 (20.7)	EPA + DHA	505	1.59	115.4	75.1	Corn oil	509	107.4	77.0	978 (96.4)	2.0
Proudman et al., 2015[58]	55.8	101 (72.7)	—	—	—	EPA + DHA	87	5.5	—	—	Sunola/ capelin oil	53	—	—	—	1.0
Raitt et al., 2005[59]	62.5	28 (14)	146 (73)	101 (50.5)	47 (23.5)	EPA + DHA	100	1.3	—	—	Olive oil	100	—	—	95 (47.5)	2.0
Risk & Prevention, 2013[60]	64	4,818 (38.5)	10577 (84.6)	10580 (84.5)	7,494 (60)	EPA + DHA	6244	0.87	150	131.8	Olive oil	6269	150	132.5	5,138 (41.1)	5.0
REDUCE-IT, 2018[4]	64	2,357 (28.8)	5,785 (70.7)	—	4,787 (58.5)	EPA	4089	4.0	216.5	74.0	Mineral oil	4090	216.0	76.0	8,145 (99.5)	4.9
SHOT, 1996[61]	59.9	79 (22)	610 (100)	137 (22.5)	—	EPA + DHA	317	3.3	—	—	No treatment	293	—		—	1.0
SCIMO, 1999[62]	58.4	44 (19.7)	223 (100)	110 (49.3)	0	EPA + DHA	112	2.0	194.7	158.3	Average European fats	111	191.2	154.4	—	2.0
SOFA, 2006[63]	61.5	85 (15.6)	320 (60)	278 (51)	87 (16)	EPA + DHA	273	0.8	—	—	Sunflower oil	273	—	—	—	1.0
SU.FOL.OM3, 2010[64]	60.7	132 (11.7)	951 (84.2)	—	—	EPA + DHA	1248	0.6	106.2	104.3	Gelatin	1253	97.4	100.4	—	4.7
Shinto et al., 2014[65]	75.5	18 (46.5)	—	—	—	EPA + DHA	13	1.65	—	—	Soybean oil	13	—	—	—	1.0
STRENGTH, 2020[6]	62.5	4,568 (35)	6,035 (46.1)	11420 (87.4)	9,170 (70.2	EPA + DHA	6539	4.0	239.0	75.0	Corn oil	6539	240.0	75.0	13,078 (100)	3.5
VITAL, 2018[5]	67.1	13,085 (50.6)	0	12884 (49.8)	3,459 (13.7)	EPA + DHA	12933	0.84	—	—	Vitamin D3	12938	—	—	8,890 (34.9)	5.3

⁎ Supplements include Vitamin C (500 mg/d), Vitamin E (400IU/d), beta-carotene (15 mg/d), zinc oxide (80 mg/d) and cupric oxide (2 mg/d). AFFORD: Multi-center Study to Evaluate the Effect of N-3 Fatty Acids [OMEGA-3] on Arrhythmia Recurrence in Atrial Fibrillation; AREDS2: Age-Related Eye Disease Study 2; ASCEND: A Study of Cardiovascular Events in Diabetes; DHA: Docosahexaenoic acid; DO IT: The Diet and Omega-3 Intervention Trial; EPIC-1: Epanova Program in Crohn's Study 1; EPE-A: Ethyl-eicosapentanoic Acid; EPA: Eicosapentaenoic acid; ENRGISE: Enabling Reduction of low-Grade Inflammation in Seniors; FAAT: Fatty Acid Antiarrhythmia Trial; FORWARD: Randomized Trial to Assess Efficacy of PUFA for the Maintenance of Sinus Rhythm in Persistent Atrial Fibrillation Fish Oil Research with omega-3 for Atrial Fibrillation Recurrence Delaying; FOSTAR: Fish oil in osteoarthritis; GISSI-P: Gruppo Italiano per lo Studio della Sopravvivenza nell'Infarto miocardico-Prevenzione; GISSI-HF: Italiano per lo Studio della Sopravvivenza nell'Infarto miocardico-Heart Failure; HEARTS: Slowing HEART Disease With Lifestyle and Omega-3 Fatty Acids trial; HARP: Heart Attach Research Program; JELIS: Japan EPA Lipid Intervention Study; LDL-C: Low-density lipoprotein-cholesterol; MAPT: Multidomain Alzheimer Prevention Trial; OFAMI: Omacor Following Acute Myocardial Infarction; ORIGIN: Outcome Reduction with an Initial Glargine Intervention; OMEMI: Omega-3 fatty acids in Elderly with Myocardial Infarction; REDUCE-IT: Reduction of Cardiovascular Events with Icosapent Ethyl-Intervention Trial; SHOT: Shunt Occlusion Trial; SCIMO: Study on Prevention of Coronary Atherosclerosis by Intervention with Marine Omega-3 fatty acids; SOFA: Study on Omega-3 Fatty Acids and Ventricular Arrhythmia Trial; SU.FOL.OM3: Supplémentation en Folates et Omega-3; STRENGTH: Long-Term Outcomes Study to Assess Statin Residual Risk with Epanova in High Cardiovascular Risk Patients with Hypertriglyceridemia; TG: triglycerides; VITAL: Vitamin D and Omega-3 Trial; *y*: years

Nineteen studies were judged at low risk of bias in all domains. All other studies had probably a high or high risk of bias in the domains of selective outcome reporting, incomplete outcome data, or blinding of participants and personnel or outcome assessment.

**Fig. 2:**
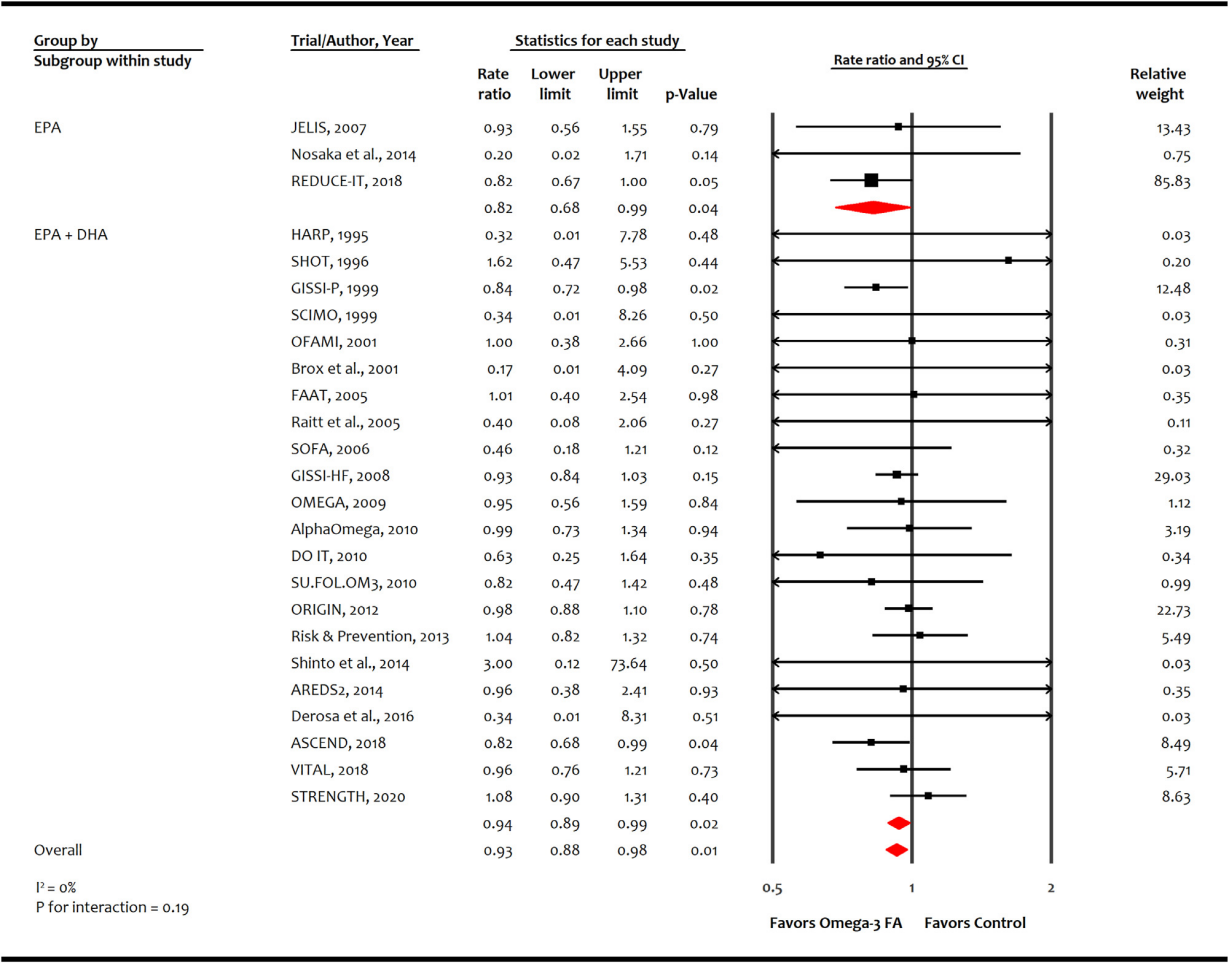
Effect of omega-3 fatty acid on cardiovascular mortality. AREDS2: Age-Related Eye Disease Study 2; ASCEND: A Study of Cardiovascular Events in Diabetes; CI: confidence interval; DHA: Docosahexaenoic acid; DO IT: The Diet and Omega-3 Intervention Trial; EPA: Eicosapentaenoic acid; FA: fatty acid; FAAT: Fatty Acid Antiarrhythmia Trial; GISSI-P: Gruppo Italiano per lo Studio della Sopravvivenza nell'Infarto miocardico-Prevenzione; GISSI-HF: Italiano per lo Studio della Sopravvivenza nell'Infarto miocardico-Heart Failure; HARP: Heart Attach Research Program; JELIS: Japan EPA Lipid Intervention Study; OFAMI: Omacor Following Acute Myocardial Infarction; ORIGIN: Outcome Reduction with an Initial Glargine Intervention; OMEMI: Omega-3 fatty acids in Elderly with Myocardial Infarction; REDUCE-IT: Reduction of Cardiovascular Events with Icosapent Ethyl-Intervention Trial; SOFA: Study on Omega-3 Fatty Acids and Ventricular Arrhythmia Trial; SU.FOL.OM3: Supplémentation en Folates et Omega-3; STRENGTH: Long-Term Outcomes Study to Assess Statin Residual Risk with Epanova in High Cardiovascular Risk Patients with Hypertriglyceridemia; TG: triglycerides; VITAL: Vitamin D and Omega-3 Trial.

A total of 25 trials (*n* = 143,514) reported 5550 events of cardiovascular mortality, and 24 trials (*n* = 140,983) reported 10,795 events of all-cause mortality. Omega-3 FA was associated with reducing cardiovascular mortality (-1.4 incident cases per 1000 person-years [-2.5, -0.4]; RR, 0.93 [0.88–0.98]; *p* = 0.01; moderate certainty; Fig. 2) but not all-cause mortality (-0.6 incident cases per 1000-person years [-1.4, 0.4]; RR, 0.97 [0.93–1.02]; *p* = 0.27; low certainty; *Appendix p 15*). Meta-analysis showed reduction in cardiovascular mortality with EPA monotherapy (RR: 0.82 [0.68–0.99]; *p* = 0.04) and EPA+DHA combination (RR: 0.94 [0.89–0.99]; *p* = 0.02) (p for interaction = 0.19).

**Fig. 3:**
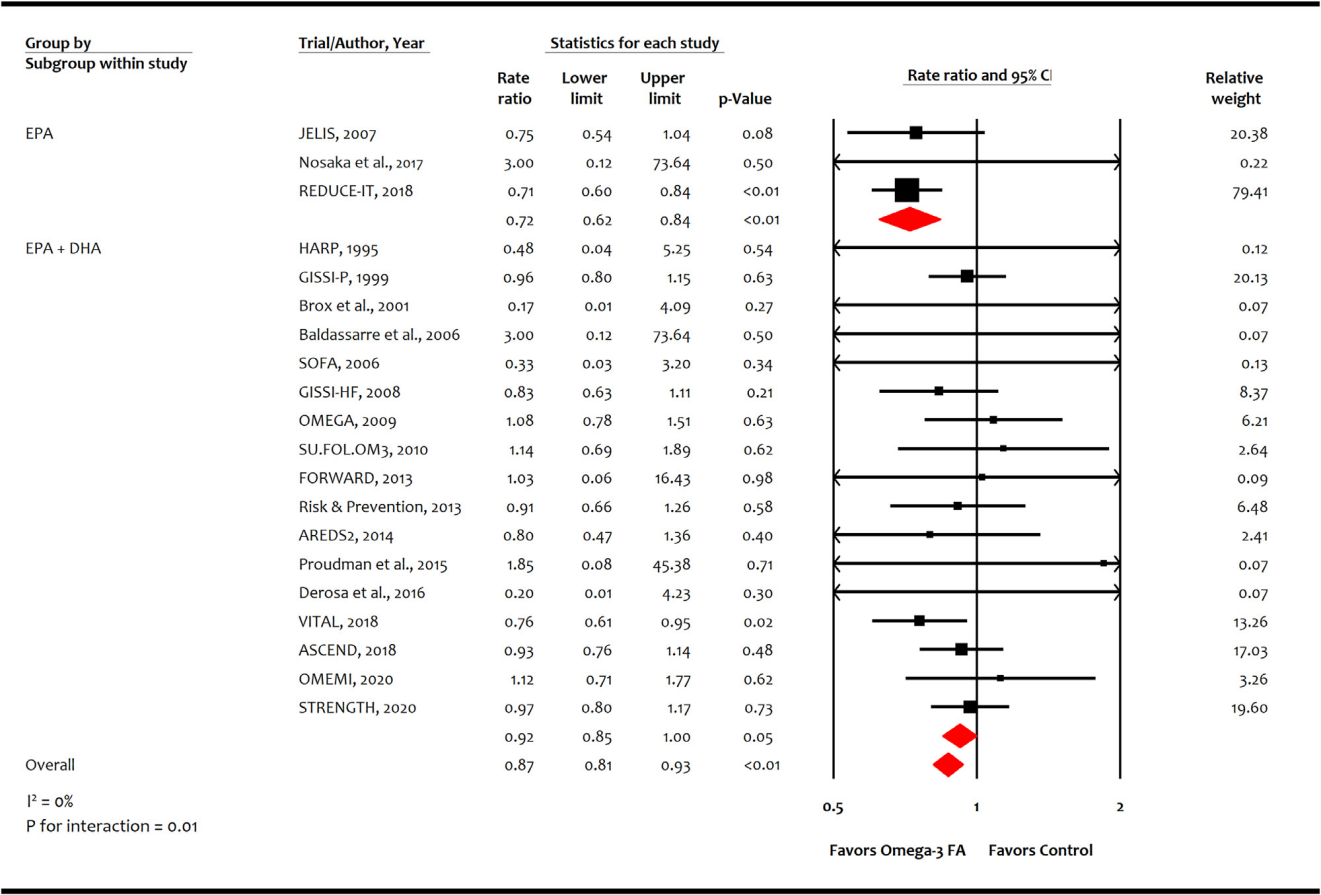
Effect of omega-3 fatty acid on non-fatal myocardial infarction. AREDS2: Age-Related Eye Disease Study 2; ASCEND: A Study of Cardiovascular Events in Diabetes; CI: confidence interval; DHA: Docosahexaenoic acid; EPA: Eicosapentaenoic acid; FA: fatty acid; FORWARD: Randomized Trial to Assess Efficacy of PUFA for the Maintenance of Sinus Rhythm in Persistent Atrial Fibrillation Fish Oil Research with omega-3 for Atrial Fibrillation Recurrence Delaying; GISSI-P: Gruppo Italiano per lo Studio della Sopravvivenza nell'Infarto miocardico-Prevenzione; GISSI-HF: Italiano per lo Studio della Sopravvivenza nell'Infarto miocardico-Heart Failure; HARP: Heart Attach Research Program; JELIS: Japan EPA Lipid Intervention Study; ORIGIN: Outcome Reduction with an Initial Glargine Intervention; OMEMI: Omega-3 fatty acids in Elderly with Myocardial Infarction; REDUCE-IT: Reduction of Cardiovascular Events with Icosapent Ethyl-Intervention Trial; SOFA: Study on Omega-3 Fatty Acids and Ventricular Arrhythmia Trial; SU.FOL.OM3: Supplémentation en Folates et Omega-3; STRENGTH: Long-Term Outcomes Study to Assess Statin Residual Risk with Epanova in High Cardiovascular Risk Patients with Hypertriglyceridemia; VITAL: Vitamin D and Omega-3 Trial.

**Fig. 4:**
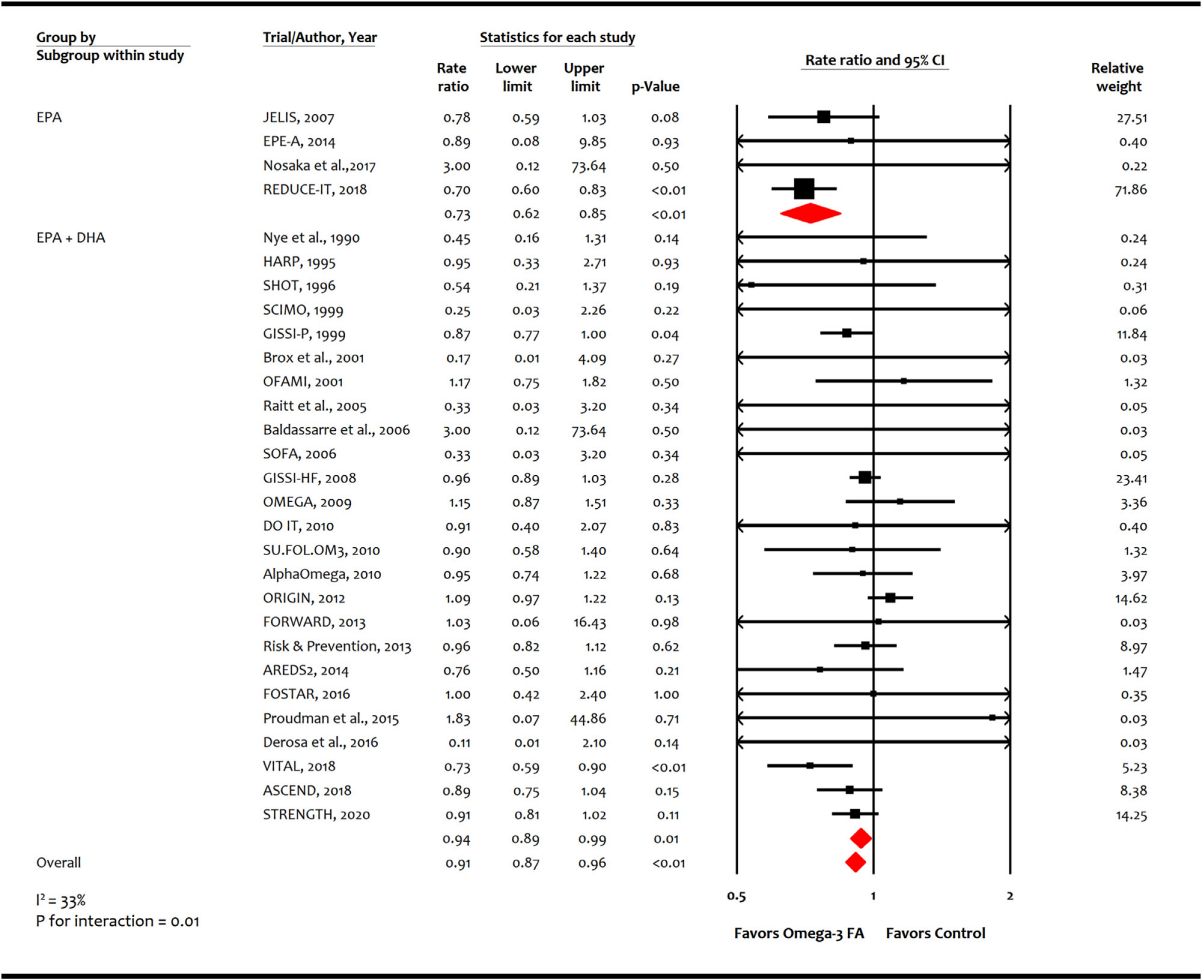
Effect of omega-3 fatty acid on coronary heart disease events. AREDS2: Age-Related Eye Disease Study 2; ASCEND: A Study of Cardiovascular Events in Diabetes; CI: confidence interval; DHA: Docosahexaenoic acid; DO IT: The Diet and Omega-3 Intervention Trial; EPE-A: Ethyl-eicosapentanoic Acid; EPA: Eicosapentaenoic acid; FORWARD: Randomized Trial to Assess Efficacy of PUFA for the Maintenance of Sinus Rhythm in Persistent Atrial Fibrillation Fish Oil Research with omega-3 for Atrial Fibrillation Recurrence Delaying; FA: fatty acid; FOSTAR: Fish oil in osteoarthritis; GISSI-P: Gruppo Italiano per lo Studio della Sopravvivenza nell'Infarto miocardico-Prevenzione; GISSI-HF: Italiano per lo Studio della Sopravvivenza nell'Infarto miocardico-Heart Failure; HARP: Heart Attach Research Program; JELIS: Japan EPA Lipid Intervention Study; LDL-C: Low-density lipoprotein-cholesterol; OFAMI: Omacor Following Acute Myocardial Infarction; ORIGIN: Outcome Reduction with an Initial Glargine Intervention; OMEMI: Omega-3 fatty acids in Elderly with Myocardial Infarction; REDUCE-IT: Reduction of Cardiovascular Events with Icosapent Ethyl-Intervention Trial; SHOT: Shunt Occlusion Trial; SCIMO: Study on Prevention of Coronary Atherosclerosis by Intervention with Marine Omega-3 fatty acids; SOFA: Study on Omega-3 Fatty Acids and Ventricular Arrhythmia Trial; SU.FOL.OM3: Supplémentation en Folates et Omega-3; STRENGTH: Long-Term Outcomes Study to Assess Statin Residual Risk with Epanova in High Cardiovascular Risk Patients with Hypertriglyceridemia; TG: triglycerides; VITAL: Vitamin D and Omega-3 Trial; *y*: years.

A total of 20 trials (*n* = 125,611) reported 2989 non-fatal MI events, and 29 trials (*n* = 144,384) reported 9153 CHD events. Omega-3 FA was associated with reducing non-fatal MI (-2.7 incident cases per 1000 person-years [-3.9, -1.4]; RR, 0.87 [0.81–0.93]; *p* = 0.0001; moderate certainty; Fig. 3) and CHD (-1.9 incident cases per 1000 person-years [-2.7, -0.8]; RR, 0.91 [0.87–0.96]; *p* = 0.0002; moderate certainty; Fig. 4). Meta-analysis showed higher risk reductions in non-fatal MI with EPA monotherapy (RR: 0.72 [0.62-0.84]; *p* = 0.00002) than EPA+DHA combination (RR: 0.92 [0.85–1.00]; p=0.05) as well as for CHD events with EPA monotherapy (RR: 0.73 [0.62–0.85]; *p* = 0.00004) than EPA+DHA combination (RR: 0.94 [0.89–0.99]; *p* = 0.01) (*p* for interaction for both endpoints = 0.01).

A total of 17 trials (*n* = 135,019) reported 13,234 events of MACE, and 13 trials (*n* = 117,890) reported 7416 events of revascularization. Omega-3 FA was associated with reducing MACE (-1.0 incident cases per 1000 person-years; RR, 0.95 [0.92–0.98]; *p* = 0.002; moderate certainty; *Appendix p 16*) and revascularization (-1.9 incident cases per 1000 person-years [-2.7, -1.0]; RR, 0.91 [0.87–0.95]; *p* = 0.0001; moderate certainty; *Appendix p 17).* Meta-analysis showed higher risk reductions in MACE with EPA monotherapy (RR: 0.78 [0.71–0.85]; *p* = 0.00000001), while EPA+DHA combination did not reduce MACE (RR: 0.99 [0.95–1.02]; *p*=0.48) (p for interaction=0.000005). This effect was consistent for revascularization.

A total of 8 trials (*n* = 65,404) reported 935 non-fatal strokes, and 8 trials (*n* = 51,336) reported 1572 events of AF. Omega-3 FA did not significantly reduce non-fatal stroke (0.8 incident cases per 1000 person-years [-1.9, 3.7]; RR, 1.04 [0.91–1.18]; *p* = 0.55; very low certainty; *Appendix p 18),* though EPA monotherapy was associated with a reduction of non-fatal stroke vs. control (RR: 0.71 [0.54–0.94]; *p* = 0.01). Conversely, omega-3 FA was associated with increased risk of AF (5.4 incident cases per 1000 person-years; RR, 1.26 [1.08–1.48]; p = 0.004; low certainty; *Appendix p 21*), with a higher risk with EPA monotherapy vs. control (RR: 1.35 [1.10–1.66]; *p* = 0.004) [4]. Overall, omega-3 FA did not prevent sudden cardiac death (*Appendix p 22*) or increase GI-related adverse events (*Appendix p 23)*, total bleeding (*Appendix p 24*), or major or minor bleeding (*Appendix p 25 and 26, respectively*); however, the meta-analysis showed a higher risk of total bleeding with EPA monotherapy vs. control (RR: 1.49 [1.20–1.84]; *p* = 0.006).

Subgroup analyses did not demonstrate heterogeneity based on age, cardiovascular disease risk, setting, or risk of bias (*Appendix p 9*). The leave-one-out meta-analysis *Appendix p 10 and 11*), or sensitivity analyses using fixed-effects model, or exclusion of older trials, trials with a high risk of bias, or trials with low cardiovascular disease risk did not influence the results (*Appendix p 12*).

## 4 Discussion

In this meta-analysis of 38 trials comprising 149,051 adult participants, we noted that omega-3 FA was associated with reducing cardiovascular mortality and other cardiovascular outcomes. Overall, trials of EPA showed higher relative reductions in cardiovascular outcomes than those of EPA+DHA, with significant interaction terms. The relative effects of omega-3 FA were consistent across all the predefined subgroups and were further supported by sensitivity analyses. While the use of omega-3 FA was not associated with a significant risk of GI-adverse events and bleeding in the overall meta-analysis, we observed a significant increase in the risk of incident AF. Moreover, EPA monotherapy was associated with higher risks of bleeding and AF; however, the evidence's certainty was low for safety outcomes.

A salient feature of this meta-analysis is the cardiovascular mortality benefit associated with the use of omega-3 FA. Although the absolute benefit was modest, we should note that it was obtained in a population with overall low baseline cardiovascular risk. Moreover, dosages of omega-3 FAs varied across the trials, with only 11% of included studies investigating ≥4 g/day omega-3 FAs. Because the magnitude of the absolute effect is a function of both baseline risk and efficacy of therapy, patients with higher baseline cardiovascular risk and those receiving higher doses of omega-3 FAs may demonstrate incremental absolute event reductions [12,13,18].

A cardiovascular survival advantage has been observed sporadically in omega-3 FA trials. The GISSI-HF (Gruppo Italiano per lo Studio della Sopravvivenza nell'Infarto miocardico-heart failure) trial showed lower total and cardiovascular mortality with omega-3 FA supplementation in patients with chronic heart failure [19]. In ASCEND, exploratory analyses showed fewer vascular deaths with omega-3 FA supplementation vs. placebo in patients with diabetes [3]. However, the most substantial scientific evidence emerged from the REDUCE-IT trial, which showed a significant 20% relative risk reduction in the pre-specified endpoint of cardiovascular death using EPA vs. placebo in patients with established ASCVD or diabetes plus other risk factors [4]. The observed cardiovascular mortality reduction in REDUCE-IT persisted in subsequent analyses of the trial [20,21].

We also noted a robust consistency in reduced cardiovascular mortality and key cardiovascular outcomes with omega-3 FA across a series of extensive sensitivity analyses. The influence analysis with stepwise exclusion of one trial at a time, including REDUCE-IT [7], did not alter the overall summary estimates. Despite the exclusion of REDUCE-IT, EPA monotherapy reduced MACE by 23% compared with the control.

The gains in life expectancy from cardiovascular causes correlate with improvement in nonfatal MI, CHD events, MACE, and revascularization [22]. The mechanistic validation was provided by the CHERRY (combination therapy of eicosapentaenoic acid and pitavastatin vs. pitavastatin alone for coronary plaque regression evaluated by intravascular ultrasonography) [23] and EVAPORATE (The Effects of Vascepa on Improving Coronary Atherosclerosis in People with High Triglycerides Taking Statin Therapy) trials [24]. In EVAPORATE, among patients with ASCVD who were taking maximally tolerated statin therapy, 4 g/day of EPA vs. placebo led to a relative reduction of 17% in low attenuation plaque volume at 18 months [24]. Since low-density plaque is independently associated with MI, these data indicate that regression of plaque with EPA can reduce ASCVD events, supporting this meta-analysis.

We noted significant variation in the relative estimates attributed to omega-3 FA formulations, with EPA trials demonstrating greater reductions in cardiovascular outcomes than those of EPA+DHA. These findings are plausible in the context of distinct biological properties not shared by EPA and DHA [1]. Various studies have shed light on the distinct structure, formation, and metabolism of EPA and DHA and their biological effects [1,8,25,26]. Although some studies have reported comparable or even greater efficacy of DHA in reducing TGs and individual pro-inflammatory cytokines than EPA [8,25,26], a significant body of evidence suggests that both EPA and DHA markedly differ regarding their effects on membrane structure, inflammatory cascade [8], lipid oxidation, endothelial function, and tissue distribution [1]. *In vivo*, omega-3 FAs (EPA and DHA) as well as omega-6 FA (arachidonic acid [AA]) are involved in formation of key regulators of inflammation, vasodilation, and platelet aggregation [26,27]. For example, the eicosanoids are produced by oxidative pathways of the cyclooxygenase (COX) and lipoxygenase (LOX) enzymes. Increased omega-6 FA intake favors high AA content in membrane phospholipids, leading to excess pro-inflammatory cytokine production through COX and LOX pathways [26,27]. In contrast, a high intake of omega-3 FA results in the increased production of EPA, which competes with AA through the same COX and LOX pathways, mitigating pro-inflammatory cytokines and generating mediators that improve vasodilation and decrease inflammation and aggregation [26,27]. Furthermore, DHA (generated after elongation, desaturation, and peroxisomal beta-oxidation of the EPA) along with EPA competes with AA for the cytochrome P450 enzymes, resulting in the production of a different set of vasodilators. These EPA- and DHA-derived moieties exert different effects on the cardiovascular system [28].

Both EPA and DHA's cellular interactions also vary based on their hydrocarbon length and number of double bonds [1]. The EPA assumes an extended conformation in lipoprotein molecules and cellular membranes, allowing it to neutralize extracellular-reactive oxygen species through its conjugated double bonds mediating stabilization of unpaired electrons [1]. Besides membrane stabilization, these functions limit oxidized LDL-C levels in plasma. In contrast, DHA possesses a longer carbon chain and one additional double bond than does EPA, which adds to membrane fluidity rather than stability. EPA can lower TGs without raising low-density lipoprotein cholesterol (LDL-C) levels, whereas DHA has been shown to increase LDL-C levels modestly in patients with elevated TG levels [[29], [30], [31]]. In this context, our findings may explain conflicting results between trials of EPA monotherapy, such as JELIS and REDUCE-IT, compared with trials of EPA+DHA, including the recent STRENGTH and OMEMI trials [6,7].

Administration of EPA reduced non-fatal stroke in REDUCE-IT and increased rates of bleeding in both JELIS and REDUCE-IT [2,4]. Rates of AF were higher with EPA in REDUCE-IT and some of the EPA+DHA trials. Therefore, the fundamental mechanisms underlying these findings merit further investigation and may provide mechanistic insights into EPA's and DHA's differential actions.

Our study has various limitations. None of the trials studied the effects of DHA monotherapy on cardiovascular outcomes. Although we performed trial-level meta-analyses, a participant-level analysis according to demographic characteristics, comorbidities, lipid profile, dosages, and baseline medical therapy would be informative. Study-level data limit us from evaluating the association of EPA or DHA levels with clinical outcomes. A systematic review reported a threshold effect suggesting that ≥250 mg of omega-3 FA per day was associated with a significant 35% reduction in sudden cardiac death.[32] In REDUCE-IT, on-treatment EPA levels were strongly correlated with the primary and secondary endpoints [33]. Similarly, a dose-response gradient between omega-3 FA and cardiovascular outcomes has been reported in previous meta-analyses [12,13]. That said, we have considered the dose-response gradient and rated the certainty of the evidence as “very serious” for indirectness during the GRADE evaluation. Another limitation of this meta-analysis is that much of the benefit seen in the pooled omega-3 FA clinical trial dataset is driven by the EPA preparation used in REDUCE-IT [4]. Furthermore, the REDUCE-IT and JELIS trials carried the highest weight in the EPA group. We could not assess the degree of non-adherence to treatment due to side effects given the scarcity of data. A total 50% of the component trials had a high risk of bias; nevertheless, subgroup and sensitivity analyses showed that study-level methodological biases did not influence the outcomes. In the case of rare events, such as all-cause mortality, survival curves tend to separate after a longer follow-up duration. Therefore, a median follow-up of 2 years across the trials might be insufficient to demonstrate a total mortality difference. For some critical endpoints, the certainty of evidence was rated very low based on the precision of estimates and indirectness derived by qualitative and quantitative heterogeneity across the trials. Finally, heterogeneity in clinical settings in the included trials might generate some concerns, although the consistency of results across subgroup and sensitivity analyses diminishes this concern.

In this systematic review and meta-analysis, we noted moderate certainty of evidence favoring omega-3 FAs for reducing cardiovascular mortality and outcomes. The magnitude of relative reductions was robust in EPA trials vs. those of EPA+DHA, suggesting differential effects of EPA and DHA in cardiovascular risk reduction. These findings also have important implications for clinical practice and treatment guidelines. After REDUCE-IT, several national and international guidelines endorsed EPA in their therapeutic recommendations [18,34]. However, the publication of two recent negative trials of EPA + DHA [6,7] has created some confusion in the scientific community about the value of omega-3 FAs in preventing ASCVD events. This meta-analysis provides reassurance about the role of omega-3 FAs, specifically EPA, in the current treatment framework of ASCVD residual cardiovascular risk reduction and encourages investigators to explore further the cardiovascular effects of EPA across different clinical settings.

## Funding

None.

## Data sharing statement

The authors declare that all supporting data are available within the article (and its online supplementary files). Any query should be submitted to the corresponding author.

## Contributors

Conception and design: S. U. Khan and D. L. Bhatt.

Analysis and interpretation of the data: S. U. Khan.

Drafting of the article: S. U. Khan, and D. L. Bhatt.

Critical revision of the article for important intellectual content: S. U. Khan, A. N. Lone, M. S. Khan, S. S. Virani, R. S. Blumenthal, K. Nasir, M. Miller, E. D. Michos, W. E. Boden, C. M. Ballantyne, D. L. Bhatt.

Final approval of the article: S. U. Khan, A. N. Lone, M. S. Khan, S. S. Virani, R. S. Blumenthal, K. Nasir, M. Miller, E. D. Michos, W. E. Boden, C. M. Ballantyne, D. L. Bhatt.

Provision of study materials or patients: S. U. Khan, A. N. Lone.

Statistical expertise: S. U. Khan.

Collection and assembly of data: S. U. Khan, A. N. Lone.

S. U. Khan is the guarantor of this work and, as such, had full access to all the data in the study and takes responsibility for the integrity of the data and the accuracy of the data analysis.

## Declaration of Competing Interest

Dr. Deepak L. Bhatt reports grants from Amarin, grants from AstraZeneca, grants from Bristol-Myers Squibb, grants from Eisai, grants from Ethicon, grants from Medtronic, grants from Sanofi Aventis, grants from The Medicines Company, unfunded research collaborations with FlowCo, grants and other from PLx Pharma, unfunded research collaborations with Takeda, personal fees from Duke Clinical Research Institute, personal fees from Mayo Clinic, personal fees from Population Health Research Institute, personal fees, non-financial support and other from American College of Cardiology, personal fees from Belvoir Publications, personal fees from Slack Publications, personal fees from WebMD, personal fees from Elsevier. Dr Bhatt is on the edvisoty board of Medscape Cardiology and Regado Biosciences, and on the borad of directoprs of Boston VA Research Institute, reports personal fees and non-financial support from Society of Cardiovascular Patient Care, non-financial support from American Heart Association, personal fees from HMP Global, grants from Roche, personal fees from Harvard Clinical Research Institute (now Baim Institute for Clinical Research), other from Clinical Cardiology, personal fees from Journal of the American College of Cardiology, other from VA, grants from Pfizer, grants from Forest Laboratories/AstraZeneca, grants from Ischemix, other from St. Jude Medical (now Abbott), other from Biotronik, grants and other from Cardax, other from Boston Scientific, grants from Amgen, grants from Lilly, grants from Chiesi, grants from Ironwood, personal fees from Cleveland Clinic, personal fees from Mount Sinai School of Medicine, other from Merck, grants from Abbott, grants from Regeneron, other from Svelte, grants and other from PhaseBio, grants from Idorsia, grants from Synaptic, personal fees from TobeSoft, grants, personal fees and other from Boehringer Ingelheim, personal fees from Bayer, grants and other from Novo Nordisk, grants from Fractyl, personal fees from Medtelligence/ReachMD, personal fees from CSL Behring, grants and other from Cereno Scientific, grants from Afimmune, grants from Ferring Pharmaceuticals, other from CSI, grants from Lexicon, personal fees from MJH Life Sciences, personal fees from Level Ex, grants from Contego Medical, grants and other from CellProthera, personal fees from K2P, personal fees from Canadian Medical and Surgical Knowledge Translation Research Group, grants and other from MyoKardia/BMS, grants from Owkin, grants from HLS Therapeutics, grants and other from Janssen, grants from 89Bio, grants and other from Novo Nordisk, grants from Garmin, grants and collaborations from Novartis, outside the submitted work.

Dr. Salim S. Virani reports grants from Department of Veterans Affairs, World Heart Federation, Tahir and Jooma Family, other from American College of Cardiology, outside the submitted work.

Dr. Michael Miller reports personal fees from Amarin, outside the submitted work.

Dr. Christie Ballantyne reports personal fees from Amarin, during the conduct of the study; grants and personal fees from Abbott Diagnostic, personal fees from AstraZeneca, grants and personal fees from Amgen, grants and personal fees from Esperion, personal fees from Matinas BioPharma, personal fees from Pfizer, grants and personal fees from Novartis, grants and personal fees from Regeneron, grants and personal fees from Roche Diagnostic, personal fees from Sanofi-Synthelabo, grants from National Institutes of Health, grants from American Heart Association, grants from American Diabetes Association, personal fees from Althera, personal fees from Novo Nordisk, grants from Akcea, personal fees from Denka Seiken, personal fees from Gilead, personal fees from Genentech, personal fees from Corvidia, personal fees from Arrowhead, personal fees from New Amsterdam, grants from Ionis, outside the submitted work.

All the other authors have nothing to disclose.

## Appendix Supplementary materials

Image, application 1
