# Supplementary material for: Effect of omega-3 fatty acids on cardiovascular outcomes: A systematic review and meta-analysis
Source: eClinicalMedicine. 2021 Jul 8;38:100997. doi: 10.1016/j.eclinm.2021.100997 (PMC8413259; doi:10.1016/j.eclinm.2021.100997)
Supplement: Supplementary file 1 [file mmc1.docx]

**Supplementary Appendix**

**Table 1.** PubMed search strategy.

| N | Search terms | Records |
| --- | --- | --- |
| 1 | *omega 3 fatty acids | 17,125 |
| 2 | *eicosapentaenoic acid | 74,399 |
| 3 | *docosahexaenoic acid | 74,399 |
| 4 | *omega 3 fatty acids AND *cardiovascular disease | 4,868 |
| 5 | *omega 3 fatty acids AND *myocardial infarction | 431 |
| 6 | *omega 3 fatty acids AND *cerebrovascular accident OR *stroke | 174 |
| 7 | *eicosapentaenoic acid AND *cardiovascular disease | 21,317 |
| 8 | *eicosapentaenoic acid AND *myocardial infarction | 1,287 |
| 9 | *eicosapentaenoic acid AND *cerebrovascular accident OR *stroke | 552 |
| 10 | *docosahexaenoic acid AND *cardiovascular disease | 21,317 |
| 11 | *docosahexaenoic acid AND *myocardial infarction | 1,287 |
| 12 | *docosahexaenoic acid AND *cerebrovascular accident OR *stroke | 552 |

**Table 2.** Risk of bias in the included trials as assessed by the Cochrane risk of bias assessment scale.

|  |  |  | **Blinding** | |  |  |  |
| --- | --- | --- | --- | --- | --- | --- | --- |
| **Trial/Author, year** | **Randomization** | **Allocation concealment** | **Participants and personnel** | **Outcome assessment** | **Selective outcome reporting** | **Incomplete outcome data** | **Other** |
| AFFORD, 2013 | Probably low risk | Probably low risk | Probably low risk | Probably low risk | High risk | Low risk | Low risk |
| AlphaOmega, 2010 | Low risk | Low risk | Low risk | Low risk | Probably high risk | Low risk | Low risk |
| AREDS2, 2014 | Low risk | Low risk | Low risk | Low risk | Low risk | Low risk | Low risk |
| ASCEND, 2018 | Low risk | Low risk | Low risk | Low risk | Low risk | Low risk | Low risk |
| Baldassarre et al., 2006 | Low risk | Probably low risk | Probably low risk | Probably low risk | Low risk | Probably low risk | Low risk |
| Brox et al., 2001 | Low risk | Low risk | High risk | High risk | Probably low risk | High risk | Low risk |
| Derosa et al., 2016 | Low risk | Low risk | Low risk | Low risk | Probably low risk | Low risk | Low risk |
| DO IT, 2010 | Low risk | Probably low risk | Probably low risk | Probably low risk | Probably low risk | Low risk | Low risk |
| ENRGISE, 2018 | Low risk | Low risk | Probably low risk | Probably low risk | High risk | High risk | Low risk |
| EPE-A, 2014 | Low risk | Low risk | Low risk | Low risk | Probably high risk | High risk | Low risk |
| EPIC-1, 2008 | Low risk | Low risk | Probably low risk | Low risk | High risk | Low risk | Low risk |
| FAAT, 2005 | Low risk | Low risk | Probably low risk | Low risk | Low risk | High risk | Low risk |
| FORWARD, 2013 | Low risk | Low risk | Low risk | Low risk | Low risk | High risk | Low risk |
| FOSTAR, 2016 | Low risk | Low risk | Low risk | Low risk | High risk | Low risk | Low risk |
| GISSI-HF, 2008 | Low risk | Low risk | Low risk | Low risk | Probably low risk | Probably low risk | Low risk |
| GISSI-P, 1999 | Low risk | Low risk | High risk | Low risk | Low risk | Low risk | Low risk |
| HARP, 1995 | Low risk | Low risk | Low risk | Low risk | High risk | Low risk | Low risk |
| HEARTS, 2017 | Low risk | Low risk | High risk | Low risk | Low risk | High risk | Low risk |
| JELIS, 2007 | Low risk | Low risk | High risk | Low risk | Low risk | Low risk | Low risk |
| Kumar et al., 2012 | Probably low risk | Probably low risk | High risk | High risk | Low risk | Low risk | Low risk |
| MAPT, 2017 | Low risk | Low risk | Low risk | Low risk | Low risk | Low risk | Low risk |
| Nye, 1990 | Probably low risk | Probably low risk | Probably low risk | Low risk | Probably low risk | Probably low risk | Low risk |
| Nosaka et al., 2017 | Low risk | Probably low risk | High risk | Probably high risk | High risk | Low risk | Low risk |
| OFAMI, 2001 | Probably low risk | Low risk | Low risk | Low risk | Probably low risk | Probably low risk | Low risk |
| OMEGA, 2009 | Low risk | Low risk | Low risk | Low risk | Low risk | Low risk | Low risk |
| OMEMI, 2020 | Low risk | Low risk | Low risk | Low risk | Probably low risk | Low risk | Low risk |
| ORIGIN, 2012 | Low risk | Low risk | Low risk | Low risk | Low risk | Low risk | Low risk |
| Proudman et al., 2015 | Low risk | Low risk | Low risk | Low risk | Probably low risk | Low risk | Low risk |
| Raitt et al., 2005 | Low risk | Probably low risk | Probably low risk | Low risk | High risk | Low risk | Low risk |
| REDUCE-IT, 2018 | Low risk | Probably low risk | Low risk | Low risk | Low risk | Low risk | Low risk |
| Risk & Prevention, 2013 | Low risk | Probably low risk | Low risk | Low risk | High risk | Low risk | Low risk |
| SCIMO, 1999 | Low risk | Low risk | Low risk | Low risk | Probably low risk | Probably low risk | Low risk |
| Shinto et al., 2014 | Low risk | Probably low risk | Low risk | Low risk | Low risk | Low risk | Low risk |
| SHOT, 1996 | Low risk | Low risk | High risk | Low risk | Probably low risk | Low risk | Low risk |
| SOFA, 2006 | Low risk | Low risk | Low risk | Low risk | Low risk | Low risk | Low risk |
| STRENGTH, 2020 | Low risk | Low risk | Low risk | Low risk | Low risk | Low risk | Low risk |
| SU.FOL.OM3, 2010 | Low risk | Low risk | Low risk | Low risk | Low risk | Low risk | Low risk |
| VITAL, 2018 | Low risk | Low risk | Low risk | Low risk | Low risk | Low risk | Low risk |

AFFORD: Multi-center Study to Evaluate the Effect of N-3 Fatty Acids [OMEGA-3] on Arrhythmia Recurrence in Atrial Fibrillation; AREDS2: Age-Related Eye Disease Study 2; ASCEND: A Study of Cardiovascular Events in Diabetes; DO IT: The Diet and Omega-3 Intervention Trial; EPIC-1: Epanova Program in Crohn’s Study 1; EPE-A: Ethyl-eicosapentanoic Acid; ENRGISE: Enabling Reduction of low-Grade Inflammation in Seniors; FAAT: Fatty Acid Antiarrhythmia Trial; FORWARD: Randomized Trial to Assess Efficacy of PUFA for the Maintenance of Sinus Rhythm in Persistent Atrial Fibrillation Fish Oil Research with omega-3 for Atrial Fibrillation Recurrence Delaying; FOSTAR: Fish oil in osteoarthritis; GISSI-P: Gruppo Italiano per lo Studio della Sopravvivenza nell’Infarto miocardico-Prevenzione; GISSI-HF: Italiano per lo Studio della Sopravvivenza nell’Infarto miocardico-Heart Failure; HEARTS: Slowing HEART Disease With Lifestyle and Omega-3 Fatty Acids trial; HARP: Heart Attach Research Program; JELIS: Japan EPA Lipid Intervention Study; MAPT: Multidomain Alzheimer Prevention Trial; OFAMI: Omacor Following Acute Myocardial Infarction; ORIGIN: Outcome Reduction with an Initial Glargine Intervention; OMEMI: Omega-3 fatty acids in Elderly with Myocardial Infarction; REDUCE-IT: Reduction of Cardiovascular Events with Icosapent Ethyl-Intervention Trial; SHOT: Shunt Occlusion Trial; SCIMO: Study on Prevention of Coronary Atherosclerosis by Intervention with Marine Omega-3 fatty acids; SOFA: Study on Omega-3 Fatty Acids and Ventricular Arrhythmia Trial; SU.FOL.OM3: Supplémentation en Folates et Omega-3; STRENGTH: Long-Term Outcomes Study to Assess Statin Residual Risk with Epanova in High Cardiovascular Risk Patients with Hypertriglyceridemia; VITAL: Vitamin D and Omega-3 Trial

**Table 3.** Baseline population and cardiovascular risk assessment at the trial level**.**

| **Trial/Author, year** | **Population** | ***CVD risk** | **Risk of bias** |
| --- | --- | --- | --- |
| AlphaOmega, 2010 | Participants, 60 - 80 years, who had had a myocardial infarction and were receiving state-of-the-art antihypertensive, antithrombotic, and lipid-modifying therapy. | High | Low |
| AFFORD, 2013 | Participants with symptomatic paroxysmal or persistent atrial fibrillation. | High | High |
| AREDS2, 2014 | Participants who were at risk for developing late age-related macular degeneration | Low | High |
| ASCEND, 2018 | Participants with diabetes but without evidence of atherosclerotic cardiovascular disease | High | High |
| Brox et al., 2001 | Subjects with moderate hypercholesterolemia. | High | High |
| Baldassare et al., 2006 | Participants with combined hyperlipoproteinemia. | High | Low |
| DO IT, 2010 | Older men with hypercholesterolemia. | High | High |
| Derosa et al., 2016 | Participants with impaired fasting glucose or impaired glucose tolerance. | Moderate | Low |
| EPIC-1, 2008 | Participants with quiescent Crohn disease. | Low | High |
| EPE-A, 2014 | Subjects with NASH and nonalcoholic fatty liver disease (NAFLD) activity scores ≥ 4, with minimum scores of 1 for steatosis and inflammation, along with either ballooning or at least stage 1a fibrosis. | Low | High |
| ENRGISE, 2018 | Participants aged 70+, with self-reported mobility impairment. | Low | High |
| FOSTAR, 2016 | Participants with knee osteoarthritis and chronic knee pain. | Low | High |
| FAAT, 2005 | Participants with implanted cardioverter/defibrillators | High | Low |
| FORWARD, 2012 | Participants with confirmed symptomatic paroxysmal AF that required cardioversion. | High | High |
| GISSI-P, 1999 | Participants surviving a recent (≤3 months) myocardial infarction. | High | Low |
| GISSI-HF, 2008 | Participants with chronic heart failure. | High | High |
| HEARTS, 2017 | Subjects with stable coronary artery disease, on statins. | High | High |
| HARP, 1995 | Participants with angiographically documented coronary heart disease and normal plasma lipid levels. | High | Low |
| JELIS, 2007 | Hypercholesterolemic participants on statins. | High | High |
| Kumar et al., 2012 | Participants with persistent atrial fibrillation | High | High |
| MAPT, 2017 | Elderly adults with memory complaints | Low | Low |
| Nye et al., 1990 | Participants undergoing percutaneous transluminal coronary angioplasty. | High | Low |
| Nosaka et al., 2017 | Participants with acute coronary syndromes. | High | High |
| OFAMI, 2001 | Participants with recent myocardial infarction. | High | High |
| OMEGA, 2009 | Participants with depression after myocardial infarction. | High | Low |
| OMEMI, 2020 | Participants aged 70-82 years old with recent (2-8 weeks) AMI | High | Low |
| ORIGIN 2012 | Participants with dysglycemia and at high risk for cardiovascular events. | Moderate | Low |
| Proudman et al., 2015 | Participants with recent-onset rheumatoid arthritis. | Low | Low |
| Raitt et al., 2005 | Participants with an implantable cardioverter-defibrillator and a recent episode of sustained ventricular tachycardia or ventricular fibrillation. | High | High |
| Risk & Prevention, 2013 | Participants with multiple cardiovascular risk factors or atherosclerotic vascular disease but not myocardial infarction. | High | High |
| REDUCE-IT, 2018 | Participants with established cardiovascular disease or diabetes and other risk factors receiving statin therapy and a fasting triglyceride level of 135 -499 mg/dl. | High | Low |
| SHOT, 1996 | Participants undergoing coronary artery bypass grafting. | High | High |
| SCIMO, 1999 | People with angiographically proven coronary artery disease. | High | High |
| SOFA, 2006 | Participants with implantable cardioverter defibrillators. | High | Low |
| SU.FOL. OM3, 2010 | Participants with coronary heart disease. | High | Low |
| Shinto et al., 2014 | Participants 55+ with a diagnosis of probable Alzheimer’s disease | Low | Low |
| STRENGTH, 2020 | Participants on a statin with high cardiovascular risk, hypertriglyceridemia, and low levels of high-density lipoprotein cholesterol (HDL-C). | High | Low |
| VITAL, 2018 | Participants without a prior history of heart disease, stroke, or cancer. | Low | Low |

* Trials with participants having a history of cardiovascular disease or those with hypercholesteremia were considered high risk for cardiovascular disease event, and those with dysglycemia were considered a moderate risk for cardiovascular disease event.^1^ CVD: cardiovascular disease. Remaining abbreviations as per webtable 2.

**Table 4.** GRADE chart for the certainty of the evidence.

| **Certainty assessment** | | | | | | | **Effect** | **Certainty** | **Importance** |
| --- | --- | --- | --- | --- | --- | --- | --- | --- | --- |
| **No. of studies** | **Study design** | **Risk of bias** | **Inconsistency** | **Indirectness** | **Imprecision** | **Other considerations** | **Relative (95% CI)** |  |  |
| **Non-fatal myocardial infarction** | | | | | | | | | |
| 20 | RCT | not serious | not serious ^a^ | very serious ^b^ | not serious ^c^ | dose-response gradient ^d^ | **RR 0.87** (0.81-0.93) | ⨁⨁⨁◯ MODERATE | Critical |
| **CHD events** | | | | | | | | | |
| 29 | RCT | not serious | not serious ^a^ | very serious ^b^ | not serious ^c^ | dose-response gradient ^d^ | **RR 0.91** (0.87-0.96) | ⨁⨁⨁◯ MODERATE | Critical |
| **MACE** | | | | | | | | | |
| 17 | RCT | not serious | not serious ^a^ | very serious ^b^ | not serious ^c^ | dose-response gradient ^d^ | **RR 0.95** (0.92-0.98) | ⨁⨁⨁◯ MODERATE | Critical |
| **Revascularization** | | | | | | | | | |
| 13 | RCT | not serious | not serious ^a^ | very serious ^b^ | not serious ^c^ | dose-response gradient ^d^ | **RR 0.91** (0.87-0.95) | ⨁⨁⨁◯ MODERATE | Critical |
| **All-cause mortality** | | | | | | | | | |
| 24 | RCT | not serious | not serious ^a^ | very serious ^b^ | serious ^c^ | dose-response gradient ^d^ | **RR 0.97** (0.93-1.02) | ⨁⨁◯◯ LOW | Critical |
| **Non-fatal stroke** | | | | | | | | | |
| 8 | RCT | not serious | very serious ^c^ | very serious ^b^ | very serious ^c^ | dose-response gradient ^d^ | **RR 1.04** (0.91-1.18) | ⨁◯◯◯ VERY LOW | Critical |
| **Cardiovascular mortality** | | | | | | | | | |
| 25 | RCT | not serious | not serious ^a^ | very serious ^b^ | not serious ^c^ | dose-response gradient ^d^ | **RR 0.93** (0.88-0.98) | ⨁⨁⨁◯ MODERATE | Critical |
| **Atrial fibrillation** | | | | | | | | | |
| 8 | RCT | not serious | not serious ^a^ | very serious ^b^ | serious ^c^ | dose-response gradient ^d^ | **RR 1.26** (1.08-1.48) | ⨁⨁◯◯ LOW | Critical |
| **GI-related adverse events** | | | | | | | | | |
| 15 | RCT | not serious | serious ^e^ | very serious ^b^ | very serious ^c^ | dose-response gradient ^d^ | **RR 1.18** (0.93 to 1.50) | ⨁◯◯◯ VERY LOW | Critical |
| **Total bleeding** | | | | | | | | | |
| 12 | RCT | not serious | serious ^e^ | very serious ^b^ | very serious ^c^ | dose-response gradient ^d^ | **RR 1.02**  (0.99 to 1.06) | ⨁◯◯◯ VERY LOW | Critical |

RCT: Randomized controlled trials; CI: Confidence interval; RR: Rate ratio

#### Explanations

a. Criteria for evaluating consistency was based on the similarity of point estimates, the extent of overlap of confidence intervals, and statistical criteria, including tests of heterogeneity and I^2^.

b. Indirectness was considered serious or very serious if cumulative evidence was derived from trials assessing interventions in participants with varying baseline cardiovascular risk and setting (primary or secondary prevention trials).

c. Imprecision was evaluated based on 95% confidence intervals’ assessment overlap with the minimally important difference for clinical benefit or harm, based on standard criteria.^2^

d. Evidence for dose-dependent gradient was extrapolated from prior studies.^3,4^

**Table 5.** Analysis stratified according to pre-defined subgroups.

|  |  | **RATE RATIO [95% CI]** | | | | | |
| --- | --- | --- | --- | --- | --- | --- | --- |
|  | **No. Of Trials** | **Non-fatal MI** | **CHD events** | **MACE** | **Revascularization** | **Cardiovascular mortality** | **All-cause mortality** |
| **Age, years** |  |  |  |  |  |  |  |
| <65 | 26 | 0.89 [0.81-0.98] | 0.90 [0.83-0.99] | 0.96 [0.89-1.03] | 0.91 [0.84-1.00] | 0.92 [0.87-0.99] | 0.98 [0.92-1.04] |
| ≥65 | 12 | 0.83 [0.71-0.97] | 0.89 [0.80-1.00] | 0.95 [0.87-1.05] | 0.86 [0.74-0.99] | 0.93 [0.85-1.02] | 0.96 [0.90-1.03] |
| *P-value* |  | 0.43 | 0.88 | 0.95 | 0.53 | 0.87 | 0.66 |
| ***Cardiovascular disease risk** | | | | | | | |
| Low/moderate | 11 | 0.76 [0.62-0.94] | 0.87 [0.66-1.14] | 0.87 [0.66-1.14] | 0.93 [0.84-1.03] | 0.98 [0.88-1.08] | 0.99 [0.92-1.07] |
| High | 27 | 0.89 [0.82-0.97] | 0.90 [0.85-0.96] | 0.90 [0.85-0.96] | 0.89 [0.81-0.99] | 0.91 [0.86-0.97] | 0.97 [0.91-1.03] |
| *P-value* |  | 0.18 | 0.80 | 0.80 | 0.56 | 0.23 | 0.61 |
| **Control** |  |  |  |  |  |  |  |
| Active | 17 | 0.86 [0.69-1.06] | 0.99 [0.93-1.05] | 0.99 [0.91-1.08] | 0.96 [0.89-1.03] | 0.96 [0.89-1.02] | 0.97 [0.92-1.03] |
| Placebo | 21 | 0.88 [0.81-0.97] | 0.85 [0.78-0.91] | 0.94 [0.87-1.02] | 0.89 [0.81-0.99] | 0.89 [0.82-0.97] | 0.98 [0.91-1.05] |
| *P-value* |  | 0.78 | <0.01 | 0.38 | 0.30 | 0.21 | 0.88 |
| ****Setting** |  |  |  |  |  |  |  |
| Primary | 22 | 0.87 [0.78-0.96] | 0.92 [0.85-0.99] | 0.96 [0.90-1.03] | 0.95 [0.89-1.00] | 0.95 [0.89-1.01] | 0.98 [0.94-1.03] |
| Secondary | 16 | 0.91 [0.78-1.06] | 0.89 [0.79-1.00] | 0.95 [0.85-1.06] | 0.86 [0.74-1.00] | 0.88 [0.80-0.97] | 0.95 [0.86-1.05] |
| *P-value* |  | 0.59 | 0.66 | 0.87 | 0.24 | 0.21 | 0.49 |
| **Risk of bias** |  |  |  |  |  |  |  |
| High | 19 | 0.90 [0.78-1.04] | 0.90 [0.82-0.98] | 0.93 [0.83-1.03] | 0.92 [0.82-1.03] | 0.90 [0.81-1.01] | 0.97 [0.86-1.09] |
| Low | 19 | 0.88 [0.78-0.96] | 0.90 [0.81-0.99] | 0.97 [0.89-1.05] | 0.91 [0.83-0.99] | 0.93 [0.88-0.99] | 0.97 [0.93-1.02] |
| *P-value* |  | 0.71 | 0.96 | 0.52 | 0.88 | 0.62 | 0.90 |

*Cardiovascular risk categorization of trials is reported in eTable 4. CI: confidence interval; MACE: Major Adverse Cardiovascular Events

** Trials with at least 60% participants with a history of cardiovascular disease were considered secondary prevention trials.^5^

**Table 6.** A leave-one-out meta-analysis on main cardiovascular outcomes.

|  | **RATE RATIO [95% CI]** | | | | | |
| --- | --- | --- | --- | --- | --- | --- |
| **Trials excluded** | **Non-fatal MI** | **CHD events** | **MACE** | **Revascularization** | **Cardiovascular mortality** | **All-cause mortality** |
| AFFORD, 2013 | ─ | ─ | 0.95 [0.89-1.01] | ─ | ─ | 0.97 [0.93-1.02] |
| AlphaOmega, 2010 | ─ | 0.90 [0.84-0.96] | 0.95 [0.89-1.01] | ─ | 0.93 [0.88-0.98] | 0.97 [0.93-1.02] |
| AREDS2, 2014 | 0.87 [0.81-0.94] | 0.90 [0.84-0.97] | 0.95 [0.89-1.02] | 0.90 [0.83-0.97] | 0.93 [0.88-0.98] |  |
| ASCEND, 2018 | 0.86 [0.80-0.93] | 0.90 [0.84-0.97] | 0.95 [0.89-1.02] | 0.89 [0.82-0.97] | 0.94 [0.89-0.99] | 0.98 [0.93-1.03] |
| Baldassarre et al., 2006 | 0.87 [0.81-0.94] | 0.90 [0.84-0.96] | ─ | ─ | ─ | ─ |
| Brox et al., 2001 | 0.87 [0.81-0.94] | 0.90 [0.84-0.96] | ─ | ─ | 0.93 [0.88-0.98] | ─ |
| Derosa et al., 2016 | 0.87 [0.81-0.94] | 0.90 [0.84-0.96] | ─ | ─ | 0.93 [0.88-0.98] | ─ |
| DO IT, 2010 | ─ | 0.90 [0.84-0.96] | ─ | ─ | 0.93 [0.88-0.98] | 0.98 [0.94-1.02] |
| EPE-A, 2014 | ─ | 0.90 [0.84-0.96] | 0.95 [0.90-1.01] | ─ | ─ | ─ |
| FAAT, 2005 | ─ | ─ | ─ | ─ | 0.93 [0.88-0.98] | 0.97 [0.93-1.02] |
| FORWARD, 2013 | 0.87 [0.81-0.94] | 0.90 [0.84-0.96] | 0.95 [0.90-1.02] | ─ | ─ | 0.97 [0.93-1.02] |
| FOSTAR, 2016 |  | 0.90 [0.84-0.96] | ─ | ─ | ─ | ─ |
| GISSI-HF, 2008 | 0.87 [0.81-0.95] | 0.89 [0.82-0.96] | ─ | ─ | 0.93 [0.87-0.99] | 0.98 [0.93-1.03] |
| GISSI-P, 1999 | 0.86 [0.79-0.92] | 0.90 [0.84-0.97] | 0.96 [0.90-1.03] | ─ | 0.94 [0.89-0.99] | 0.98 [0.95-1.02] |
| HARP, 1995 | 0.87 [0.81-0.94] | 0.90 [0.84-0.96] | ─ | ─ | 0.93 [0.88-0.98] |  |
| JELIS, 2007 | 0.88 [0.81-0.94] | 0.91 [0.85-0.97] | 0.96 [0.91-1.03] | 0.91 [0.84-0.99] | 0.93 [0.88-0.98] | 0.97 [0.93-1.01] |
| MAPT, 2017 | ─ | ─ | ─ | ─ | ─ | 0.97 [0.93-1.02] |
| Nye, 1990 | ─ | 0.90 [0.84-0.96] | ─ | ─ | ─ | ─ |
| Nosaka et al., 2017 | 0.87 [0.81-0.94] | 0.90 [0.84-0.96] | 0.96 [0.90-1.02] | 0.91 [0.84-0.98] | 0.93 [0.88-0.98] | 0.97 [0.94-1.01] |
| OFAMI, 2001 | ─ | 0.90 [0.84-0.96] | ─ | 0.91 [0.84-0.98] | 0.93 [0.88-0.98] | 0.97 [0.93-1.02] |
| OMEGA, 2009 | 0.86 [0.80-0.93] | 0.89 [0.83-0.95] | 0.94 [0.89-1.00] | 0.90 [0.83-0.98] | 0.93 [0.88-0.98] | 0.97 [0.93-1.01] |
| OMEMI, 2020 | 0.86 [0.80-0.93] | ─ | 0.95 [0.89-1.01] | 0.91 [0.84-0.98] |  | 0.97 [0.93-1.02] |
| ORIGIN, 2012 | ─ | 0.88 [0.83-0.94] | 0.95 [0.89-1.01] | 0.90 [0.82-0.98] | 0.91 [0.86-0.97] | 0.97 [0.93-1.02] |
| Proudman et al., 2015 | 0.87 [0.81-0.94] | 0.90 [0.84-0.96] | ─ | ─ | ─ | ─ |
| Raitt et al., 2005 | ─ | 0.90 [0.84-0.96] | ─ | ─ | 0.93 [0.88-0.98] | 0.97 [0.94-1.02] |
| REDUCE-IT, 2018 | 0.91 [0.84-0.99] | 0.93 [0.88-0.98] | 0.97 [0.93-1.02] | 0.95 [0.90-0.99] | 0.94 [0.89-0.99] | 0.98 [0.94-1.02] |
| Risk & Prevention, 2013 | 0.87 [0.80-0.94] | 0.89 [0.83-0.96] | 0.95 [0.89-1.02] | 0.90 [0.83-0.98] | 0.92 [0.87-0.97] | 0.97 [0.93-1.02] |
| SCIMO, 1999 | ─ | 0.90 [0.84-0.96] | ─ | ─ | 0.93 [0.88-0.98] | 0.97 [0.93-1.02] |
| Shinto et al., 2014 | ─ |  | ─ | ─ | 0.93 [0.88-0.98] | 0.97 [0.93-1.02] |
| SHOT, 1996 | ─ | 0.90 [0.84-0.97] | ─ | ─ | 0.93 [0.88-0.98] | ─ |
| SOFA, 2006 | 0.87 [0.81-0.94] | 0.90 [0.84-0.96] | ─ | ─ | 0.93 [0.88-0.98] | 0.98 [0.93-1.02] |
| STRENGTH, 2020 | 0.85 [0.79-0.92] | 0.90 [0.83-0.97] | 0.95 [0.89-1.02] | 0.90 [0.83-0.98] | 0.92 [0.87-0.97] | 0.96 [0.93-1.00] |
| SU.FOL.OM3, 2010 | 0.87 [0.80-0.93] | 0.90 [0.84-0.96] | 0.95 [0.89-1.01] | 0.90 [0.83-0.98] | 0.93 [0.88-0.98] | 0.97 [0.93-1.02] |
| VITAL, 2018 | 0.88 [0.82-0.95] | 0.91 [0.86-0.98] | 0.96 [0.90-1.02] | 0.91 [0.84-0.99] | 0.93 [0.88-0.98] | 0.97 [0.93-1.02] |

Dash: outcome was not reported by trial. Remaining abbreviations as per webtable 2.

**Table 7.** Influence of pre-specified sensitivity analysis on outcomes.

|  | **RATE RATIO [95% CI]** | | | | | |
| --- | --- | --- | --- | --- | --- | --- |
| **Analyses** | **Non-fatal MI** | **CHD events** | **MACE** | **Revascularization** | **Cardiovascular mortality** | **All-cause mortality** |
| **Fixed-effects model** |  |  |  |  |  |  |
| EPA | 0.72 [0.62-0.84] | 0.72 [0.63-0.83] | 0.78 [0.72-0.85] | 0.74 [0.66-0.82] | 0.82 [0.68-0.99] | 0.97 [0.86-1.08] |
| EPA+DHA | 0.92 [0.85-1.00] | 0.94 [0.90-0.98] | 0.99 [0.95-1.02] | 0.95 [0.91-1.00] | 0.94 [0.89-0.99] | 0.97 [0.93-1.01] |
| Over all | 0.87 [0.81-0.93] | 0.92 [0.88-0.96] | 0.95 [0.92-0.98] | 0.91 [0.87-0.95] | 0.93 [0.88-0.98] | 0.97 [0.94-1.01] |
| **Exclusion of trials published before 2000** | | | | | | |
| EPA | 0.72 [0.62-0.84] | 0.72 [0.63-0.83] | 0.78 [0.72-0.85] | 0.75 [0.63-0.90] | 0.82 [0.68-0.99] | 0.94 [0.72-1.22] |
| EPA+DHA | 0.91 [0.83-1.00] | 0.95 [0.89-1.01] | 1.00 [0.96-1.04] | 0.95 [0.91-1.00] | 0.95 [0.90-1.01] | 0.99 [0.95-1.03] |
| Over all | 0.86 [0.79-0.93] | 0.91 [0.86-0.96] | 0.96 [0.92-0.99] | 0.94 [0.89-0.98] | 0.94 [0.89-0.99] | 0.99 [0.94-1.03] |
| **Exclusion of trials with a high risk of bias** | | | | | | |
| EPA | 0.71 [0.60-0.84] | 0.70 [0.60-0.83] | 0.78 [0.71-0.86] | 0.69 [0.61-0.79] | 0.82 [0.67-1.00] | 0.88 [0.75-1.04] |
| EPA+DHA | 0.91 [0.83-1.01] | 0.94 [0.86-1.02] | 1.00 [0.95-1.04] | 0.95 [0.90-1.00] | 0.95 [0.89-1.01] | 0.98 [0.94-1.03] |
| Over all | 0.86 [0.79-0.93] | 0.88 [0.82-0.95] | 0.96 [0.92-1.00] | 0.91 [0.87-0.96] | 0.93 [0.88-0.99] | 0.97 [0.93-1.02] |
| **Exclusion of trials with low cardiovascular disease risk** | | | | | | |
| EPA | 0.72 [0.62-0.84] | 0.72 [0.63-0.83] | 0.78 [0.72-0.85] | 0.75 [0.63-0.90] | 0.82 [0.68-0.99] | 0.94 [0.72-1.22] |
| EPA+DHA | 0.95 [0.87-1.04] | 0.93 [0.89-0.98] | 0.99 [0.95-1.04] | 0.96 [0.90-1.03] | 0.92 [0.86-0.98] | 0.97 [0.92-1.02] |
| Over all | 0.89 [0.82-0.96] | 0.91 [0.87-0.95] | 0.94 [0.90-0.98] | 0.94 [0.88-0.99] | 0.91 [0.86-0.97] | 0.97 [0.92-1.02] |
| **Exclusion of REDUCE-IT** |  |  |  |  |  |  |
| EPA | 0.76 [0.55-1.05] | 0.79 [0.59-1.05] | 0.77 [0.66-0.91] | 0.84 [0.70-1.02] | 0.86 [0.53-1.41] | 1.06 [0.88-1.26] |
| EPA+DHA | 0.92 [0.85-1.00] | 0.94 [0.89-0.99] | 0.99 [0.95-1.03] | 0.95 [0.91-1.00] | 0.94 [0.89-0.99] | 0.98 [0.93-1.02] |
| Over all | 0.91 [0.84-0.99] | 0.93 [0.88-0.98] | 0.97 [0.94-1.01] | 0.95 [0.90-0.99] | 0.93 [0.88-0.99] | 0.98 [0.94-1.03] |

CI: confidence interval; MACE: Major Adverse Cardiovascular Events.

**Table 8.** Data on events for main outcomes from trials.

| **Trial/Author, year** | **Non-fatal MI**  (125,611 participants) | | **CHD events**  (144,384 participants) | | **MACE**  (134,776 participants) | | **Revascularization**  (117,890 participants) | | **Cardiovascular mortality**  (143,514 participants) | | **All-cause mortality**  (140,983 participants) | |
| --- | --- | --- | --- | --- | --- | --- | --- | --- | --- | --- | --- | --- |
|  | **Active** | **Control** | **Active** | **Control** | **Active** | **Control** | **Active** | **Control** | **Active** | **Control** | **Active** | **Control** |
| AFFORD, 2013 | ─ | ─ |  |  | 20 | 11 | ─ | ─ | ─ | ─ | 0 | 1 |
| AlphaOmega, 2010 | ─ | ─ | 120 | 128 | 336 | 335 | ─ | ─ | 80 | 82 | 186 | 184 |
| AREDS2, 2014 | 25 | 30 | 39 | 49 | 183 | 187 | 60 | 53 | 9 | 9 |  |  |
| ASCEND, 2018 | 186 | 200 | 275 | 310 | 882 | 887 | 368 | 356 | 196 | 240 | 752 | 788 |
| Baldassarre et al., 2006 | 1 | 0 | 1 | 0 | ─ | ─ | ─ | ─ | ─ | ─ | ─ | ─ |
| Brox et al., 2001 | 0 | 1 | 0 | 1 | ─ | ─ | ─ | ─ | 0 | 1 | ─ | ─ |
| Derosa et al., 2016 | 0 | 2 | 0 | 4 | ─ | ─ | ─ | ─ | 0 | 1 | ─ | ─ |
| DO IT, 2010 |  |  | 11 | 12 | ─ | ─ | ─ | ─ | 7 | 11 | 14 | 24 |
| EPE-A, 2014 | ─ | ─ | 2 | 1 | 5 | 6 | ─ | ─ | ─ | ─ | ─ | ─ |
| FAAT, 2005 | ─ | ─ | ─ | ─ | ─ | ─ | ─ | ─ | 9 | 9 | 12 | 13 |
| FORWARD, 2013 | 1 | 1 | 1 | 1 | 16 | 20 | ─ | ─ | ─ | ─ | 4 | 5 |
| FOSTAR, 2016 | ─ | ─ | 10 | 10 | ─ | ─ | ─ | ─ | ─ | ─ | ─ | ─ |
| GISSI-HF, 2008 | 87 | 104 | 1309 | 1360 | ─ | ─ | ─ | ─ | 712 | 765 | 955 | 1014 |
| GISSI-P, 1999 | 223 | 233 | 424 | 485 | 715 | 785 | ─ | ─ | 291 | 348 | 472 | 545 |
| HARP, 1995 | 1 | 2 | 7 | 7 | ─ | ─ | ─ | ─ | 0 | 1 | ─ | ─ |
| JELIS, 2007 | 62 | 83 | 88 | 113 | 262 | 324 | 191 | 222 | 29 | 31 | 286 | 265 |
| MAPT, 2017 | ─ | ─ | ─ | ─ | ─ | ─ | ─ | ─ | ─ | ─ | 0 | 2 |
| Nye, 1990 | ─ | ─ | 5 | 11 | ─ | ─ | ─ | ─ | ─ | ─ | ─ | ─ |
| Nosaka et al., 2017 | 1 | 0 | 1 | 0 | 14 | 28 | 9 | 15 | 1 | 5 | 2 | 9 |
| OFAMI, 2001 | ─ | ─ | 42 | 36 | ─ | ─ | 43 | 49 | 8 | 8 | 11 | 11 |
| OMEGA, 2009 | 74 | 67 | 112 | 96 | 182 | 149 | 466 | 482 | 28 | 29 | 88 | 70 |
| OMEMI, 2020 | 39 | 35 | ─ | ─ | 108 | 102 | 14 | 21 | ─ | ─ | 28 | 28 |
| ORIGIN, 2012 | ─ | ─ | 635 | 580 | 1034 | 1017 | 866 | 896 | 574 | 581 | 951 | 964 |
| Proudman et al., 2015 | 1 | 0 | 1 | 0 | ─ | ─ | ─ | ─ | ─ | ─ | ─ | ─ |
| Raitt et al., 2005 | ─ | ─ | 1 | 3 | ─ | ─ | ─ | ─ | 2 | 5 | 4 | 10 |
| REDUCE-IT, 2018 | 237 | 332 | 250 | 355 | 705 | 901 | 376 | 544 | 174 | 213 | 274 | 310 |
| Risk & Prevention, 2013 | 70 | 77 | 310 | 324 | 733 | 745 | 334 | 347 | 142 | 137 | 348 | 337 |
| SCIMO, 1999 | ─ | ─ | 1 | 4 | ─ | ─ | ─ | ─ | 0 | 1 | 1 | 2 |
| Shinto et al., 2014 | ─ | ─ | ─ | ─ | ─ | ─ | ─ | ─ | 1 | 0 | 1 | 1 |
| SHOT, 1996 | ─ | ─ | 7 | 12 | ─ | ─ | ─ | ─ | 7 | 4 | ─ | ─ |
| SOFA, 2006 | 1 | 3 | 1 | 3 | ─ | ─ | ─ | ─ | 6 | 13 | 8 | 14 |
| STRENGTH, 2020 | 218 | 226 | 556 | 616 | 785 | 795 | 414 | 441 | 228 | 211 | 373 | 333 |
| SU.FOL.OM3, 2010 | 32 | 28 | 37 | 41 | 81 | 76 | 152 | 156 | 23 | 28 | 58 | 59 |
| VITAL, 2018 | 132 | 174 | 145 | 200 | 386 | 419 | 247 | 294 | 142 | 148 | 493 | 485 |

Abbreviations as per webtable 2.


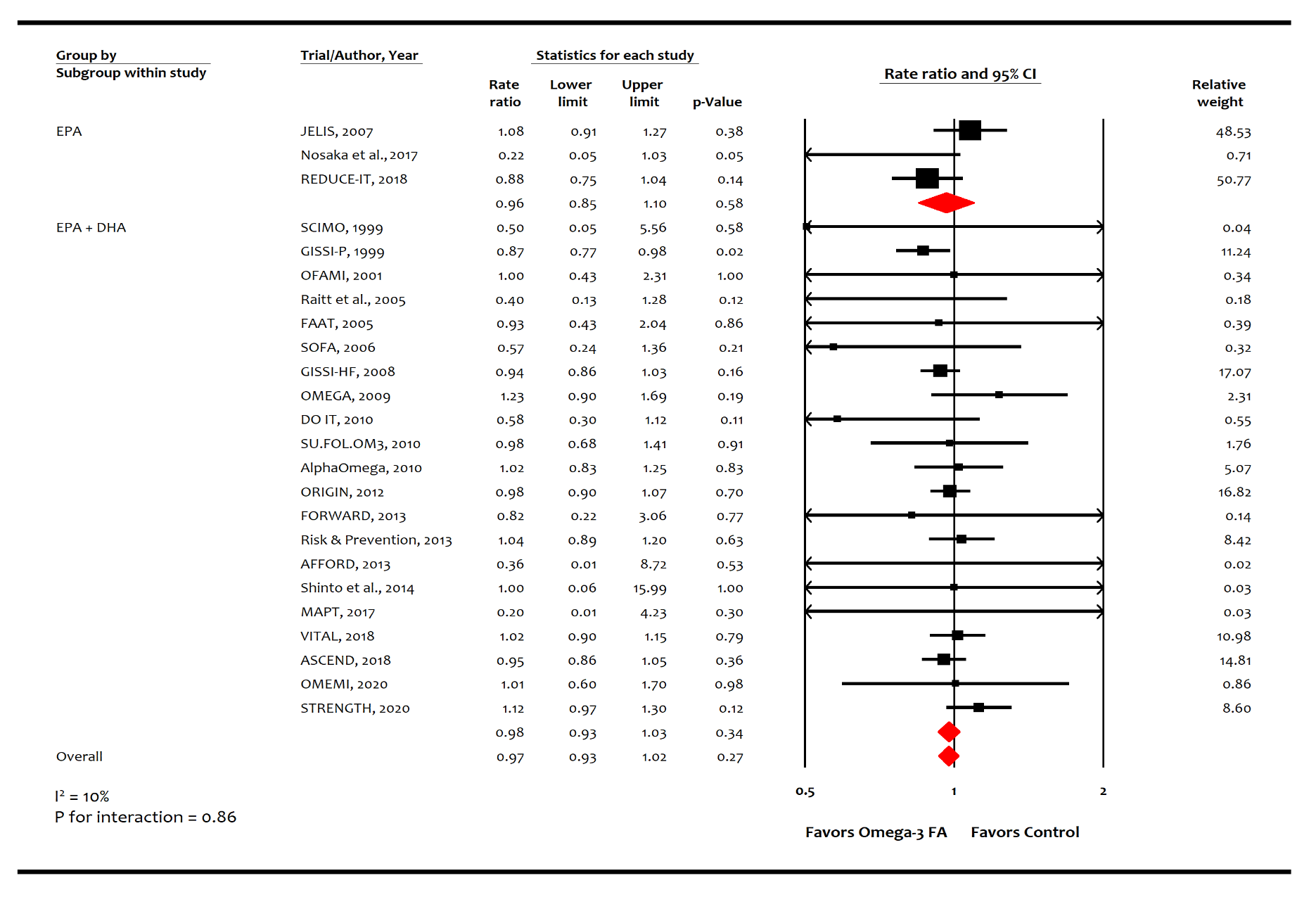
**Figure 1.** Effect of omega-3 fatty acid on all-cause mortality.


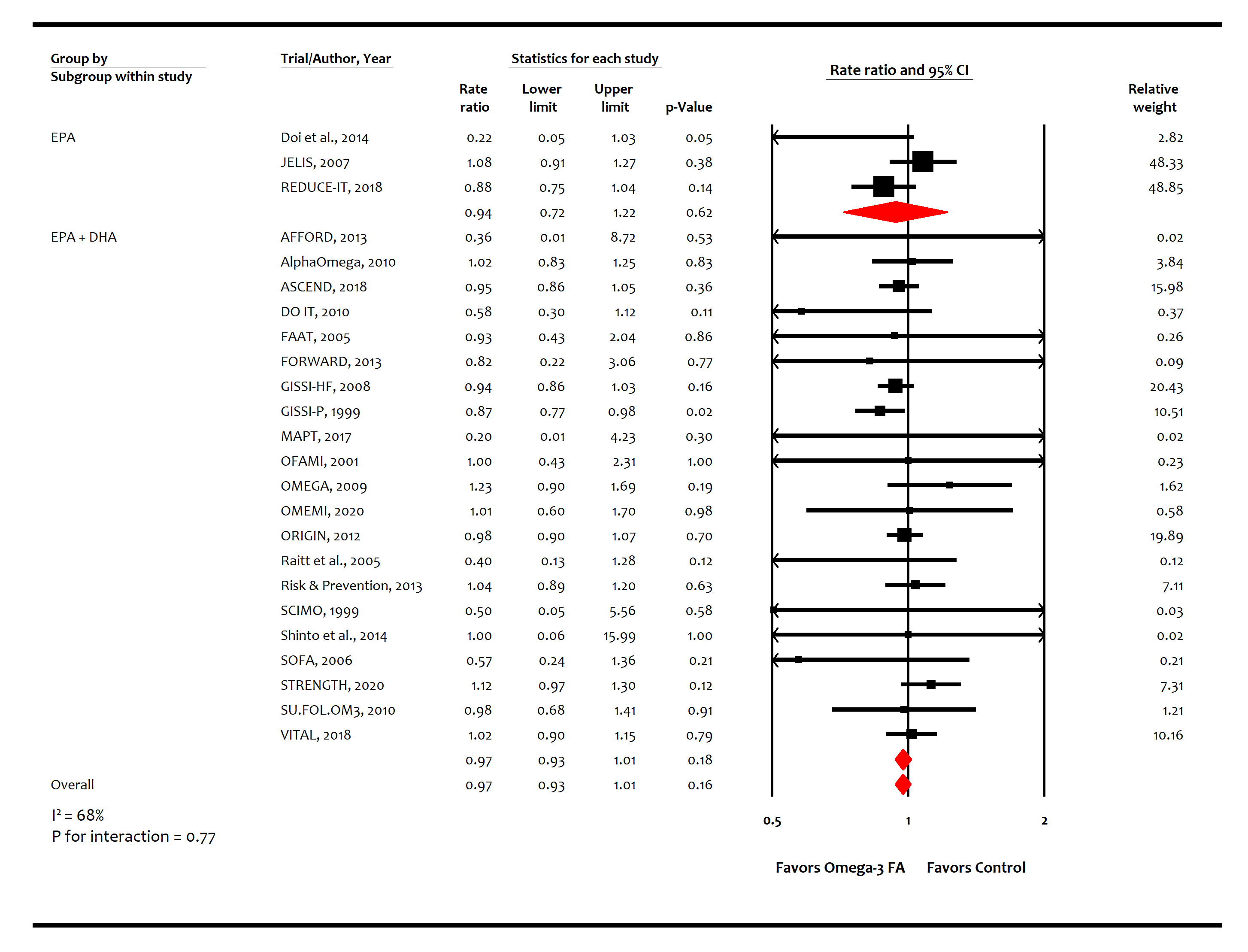


CI: confidence interval; DHA: docosahexaenoic acid; EPA: eicosapentaenoic acid; FA: fatty acid. Remaining abbreviations as per webtable 2.


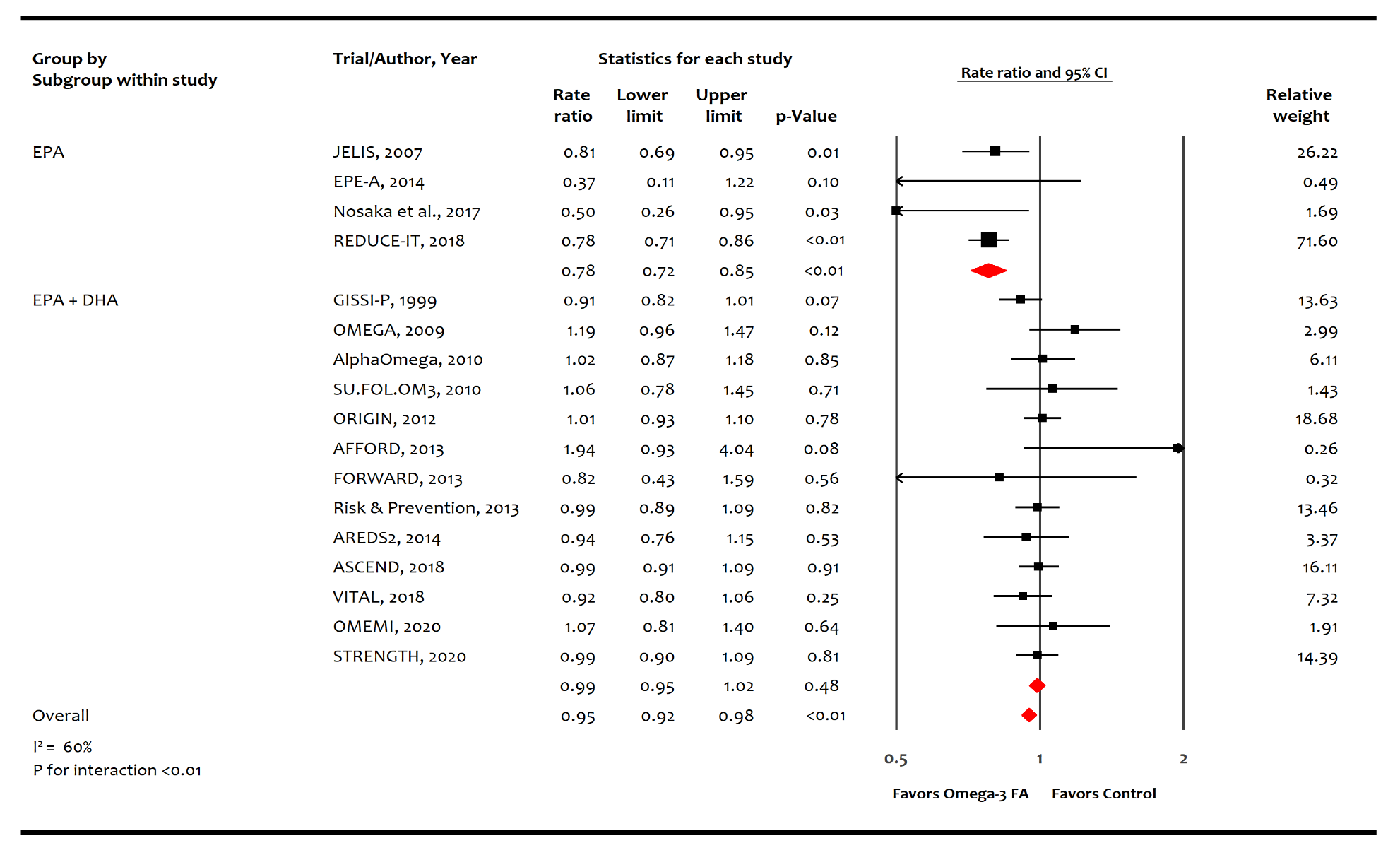
**Figure 2.** Effect of omega-3 fatty acid on major adverse cardiovascular events (MACE).

CI: confidence interval; DHA: docosahexaenoic acid; EPA: eicosapentaenoic acid; FA: fatty acid. Remaining abbreviations as per webtable 2.


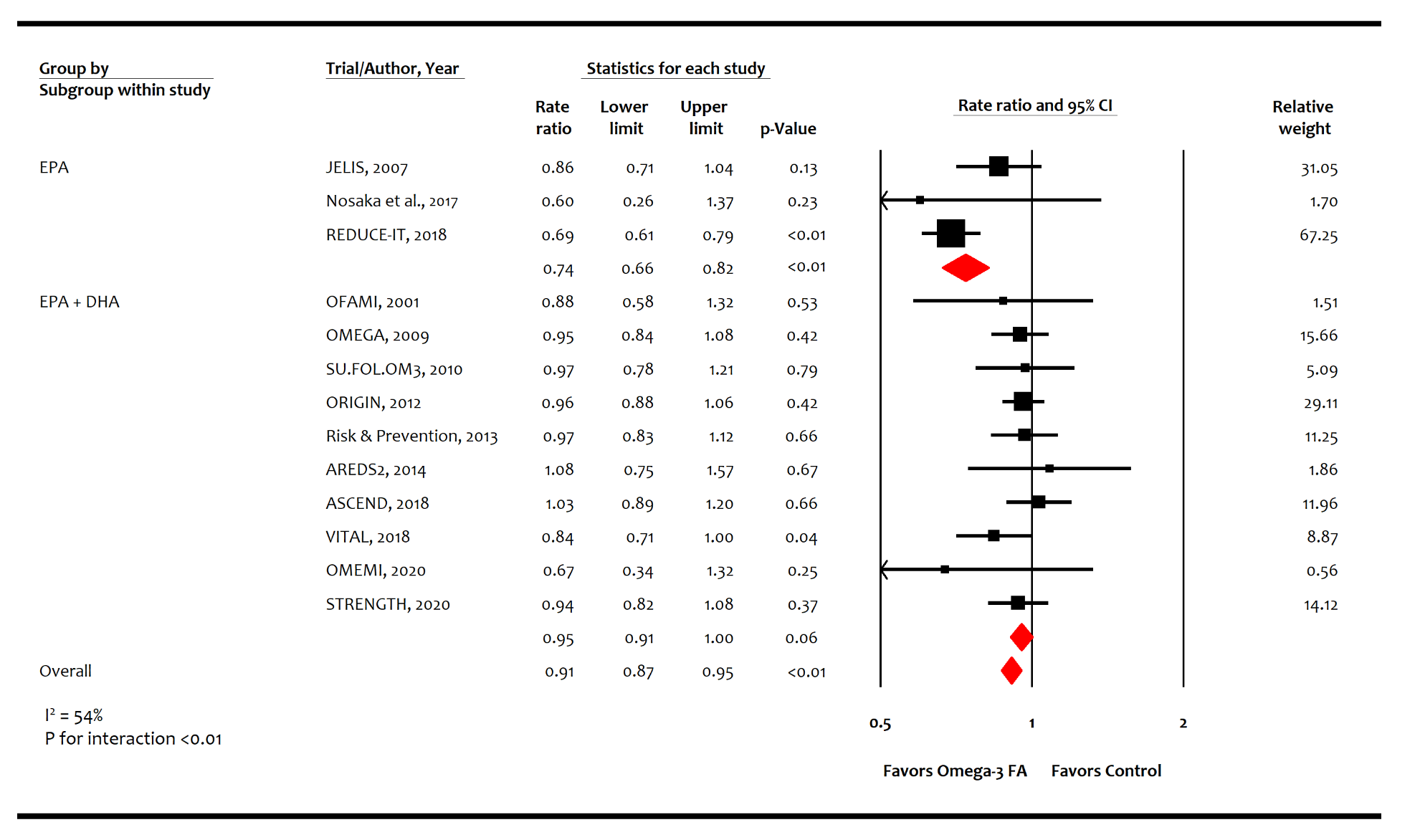
**Figure 3.** Effect of omega-3 fatty acid on revascularization.

CI: confidence interval; DHA: docosahexaenoic acid; EPA: eicosapentaenoic acid; FA: fatty acid. Remaining abbreviations as per webtable 2.

**Figure 4.** Effect of omega-3 fatty acid on non-fatal stroke.


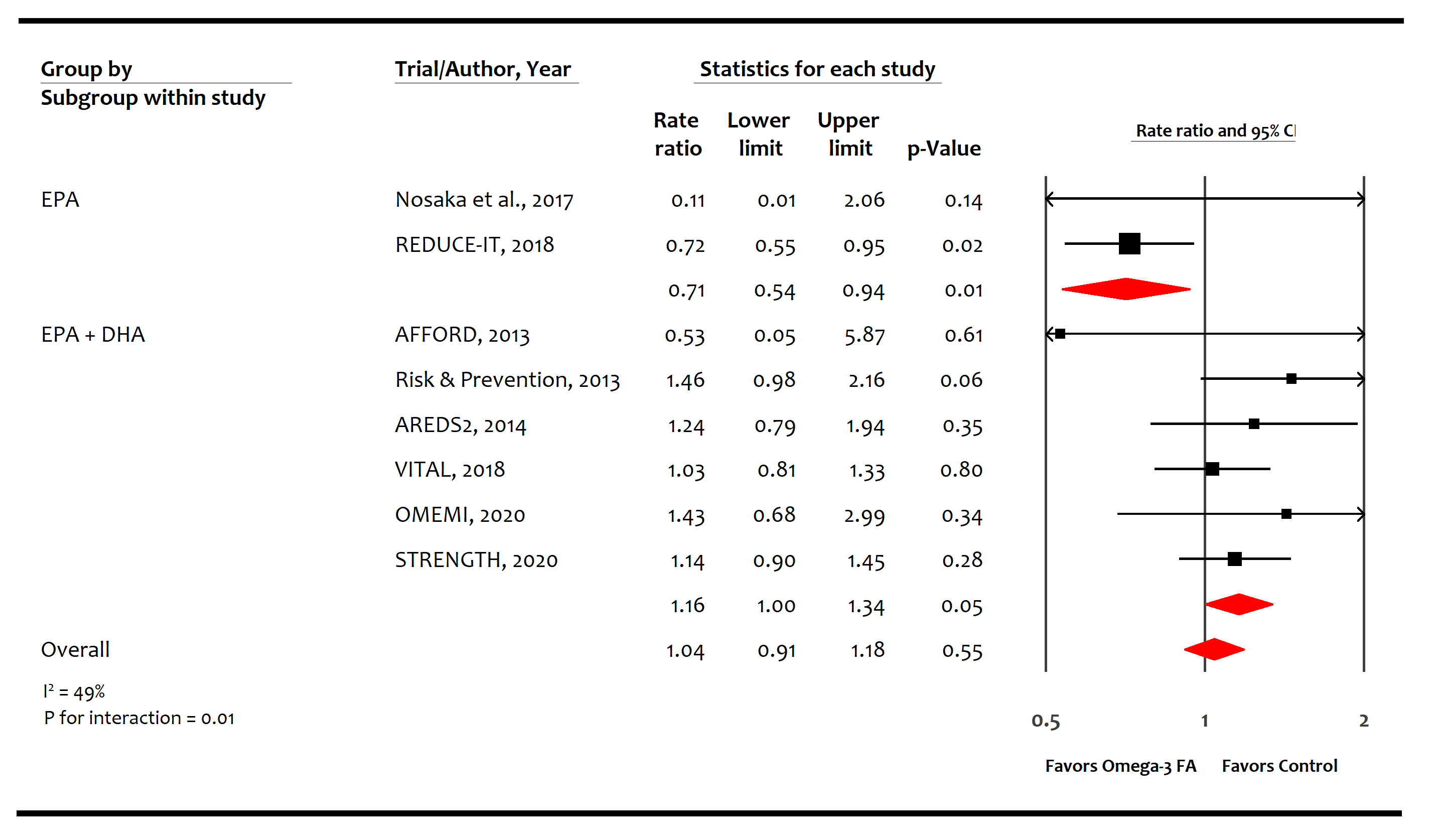


CI: confidence interval; FA: fatty acid. Remaining abbreviations as per webtable 2.

**
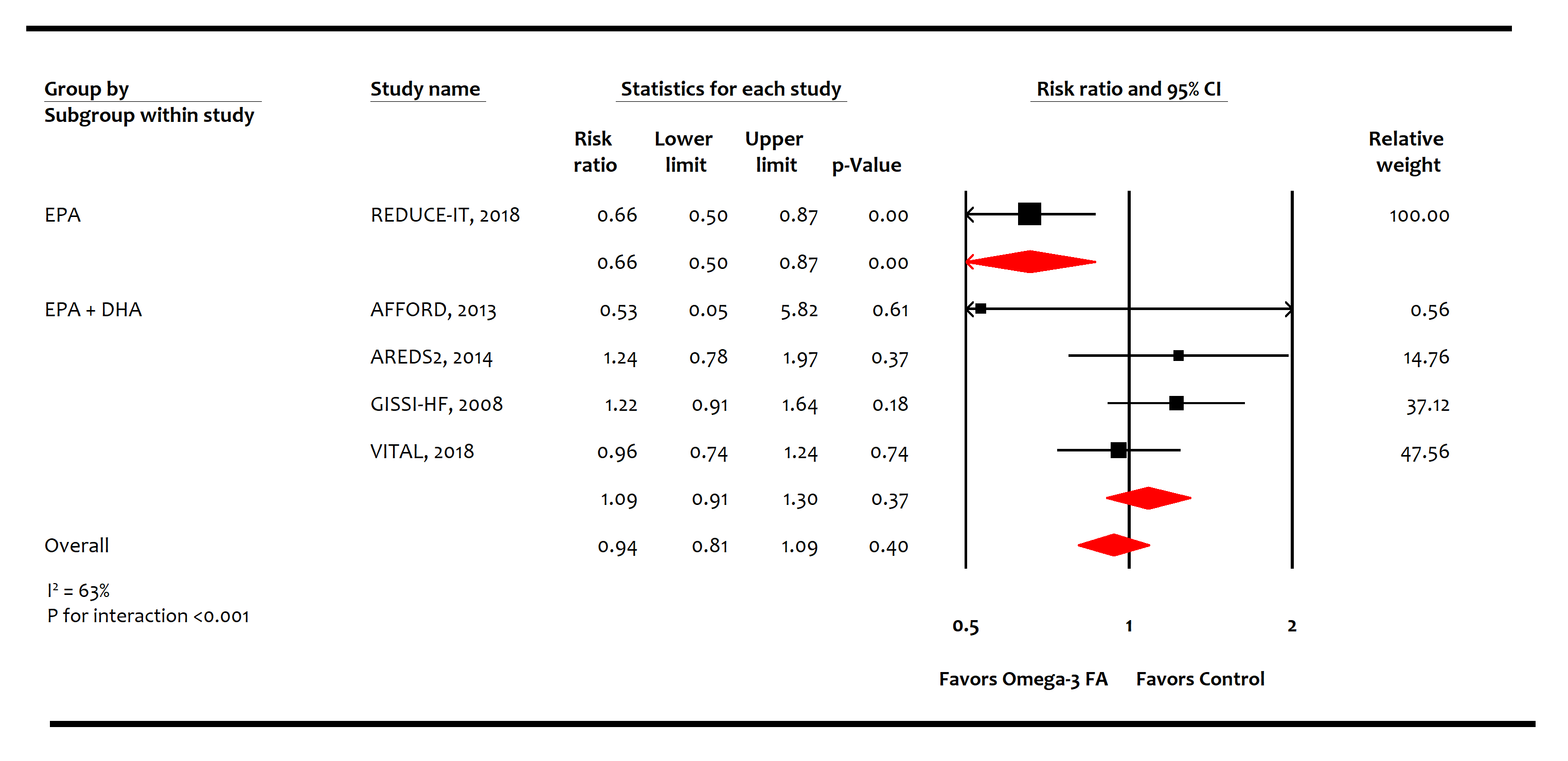
Figure 5.** Effect of omega-3 fatty acid on ischemic stroke.

CI: confidence interval; FA: fatty acid. Remaining abbreviations as per webtable 2.

**
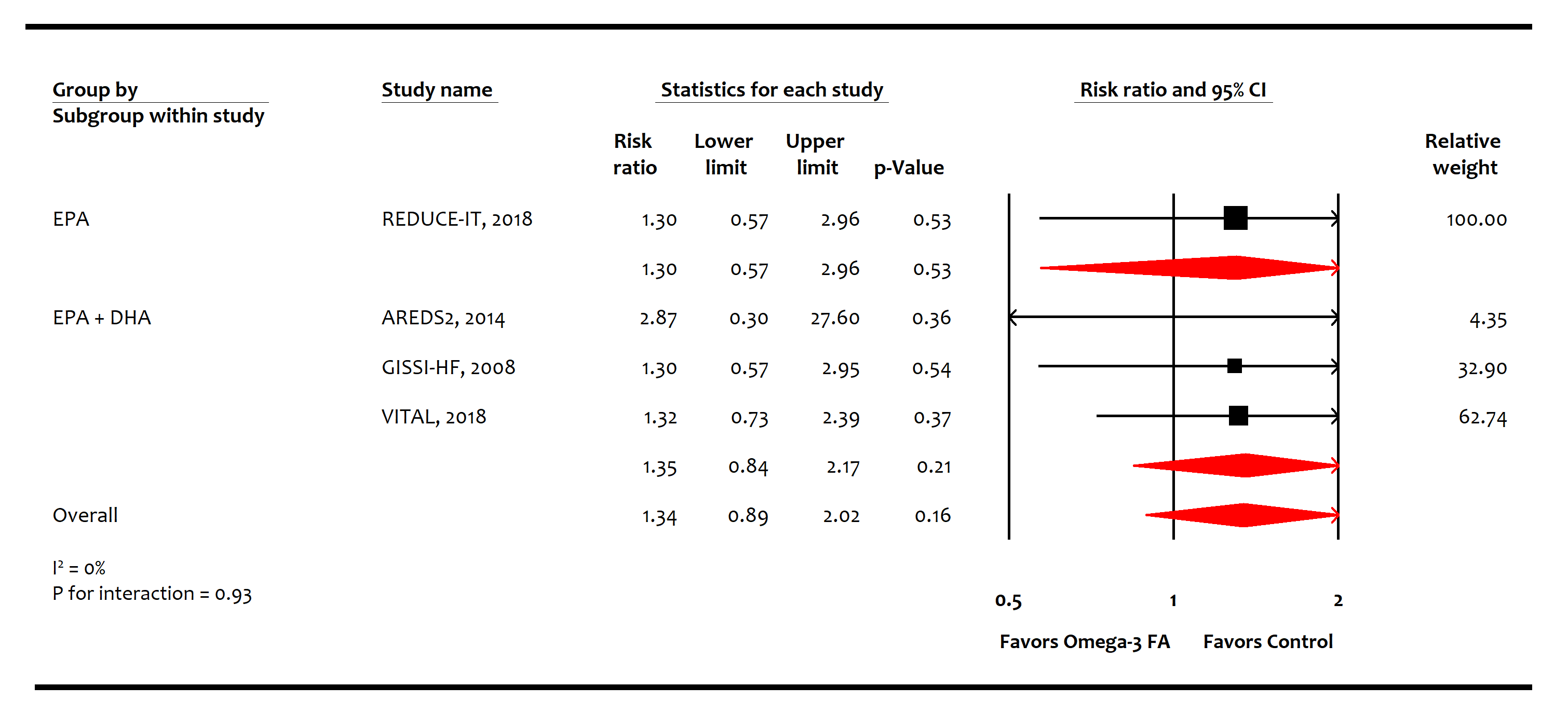
Figure 6.** Effect of omega-3 fatty acid on hemorrhagic stroke

CI: confidence interval; FA: fatty acid. Remaining abbreviations as per webtable 2.


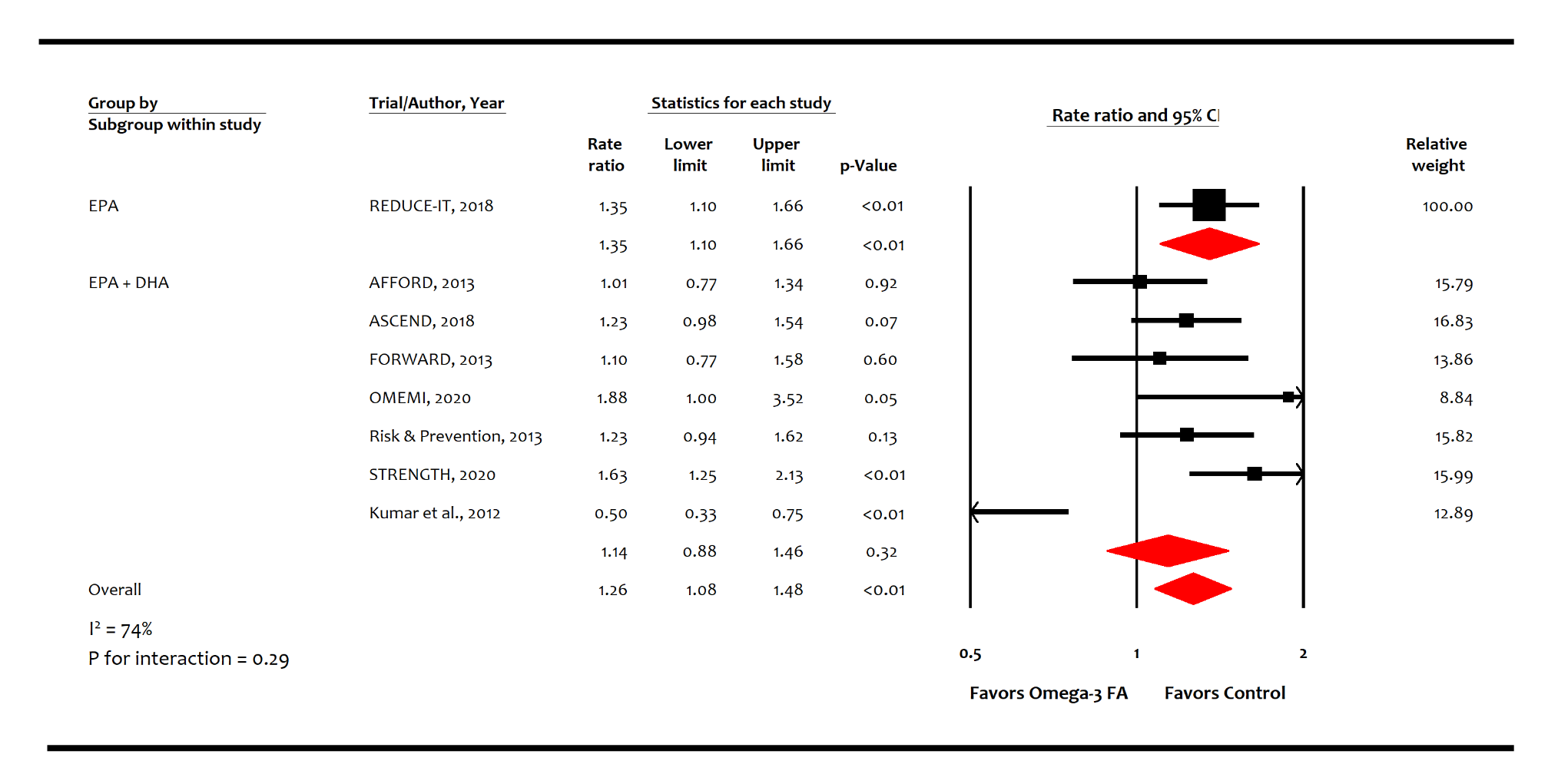
**Figure 7.** Effect of omega-3 fatty acid on atrial fibrillation.

CI: confidence interval; FA: fatty acid. Remaining abbreviations as per webtable 2.

**Figure 8.** Effect of omega-3 fatty acid on sudden cardiac death.


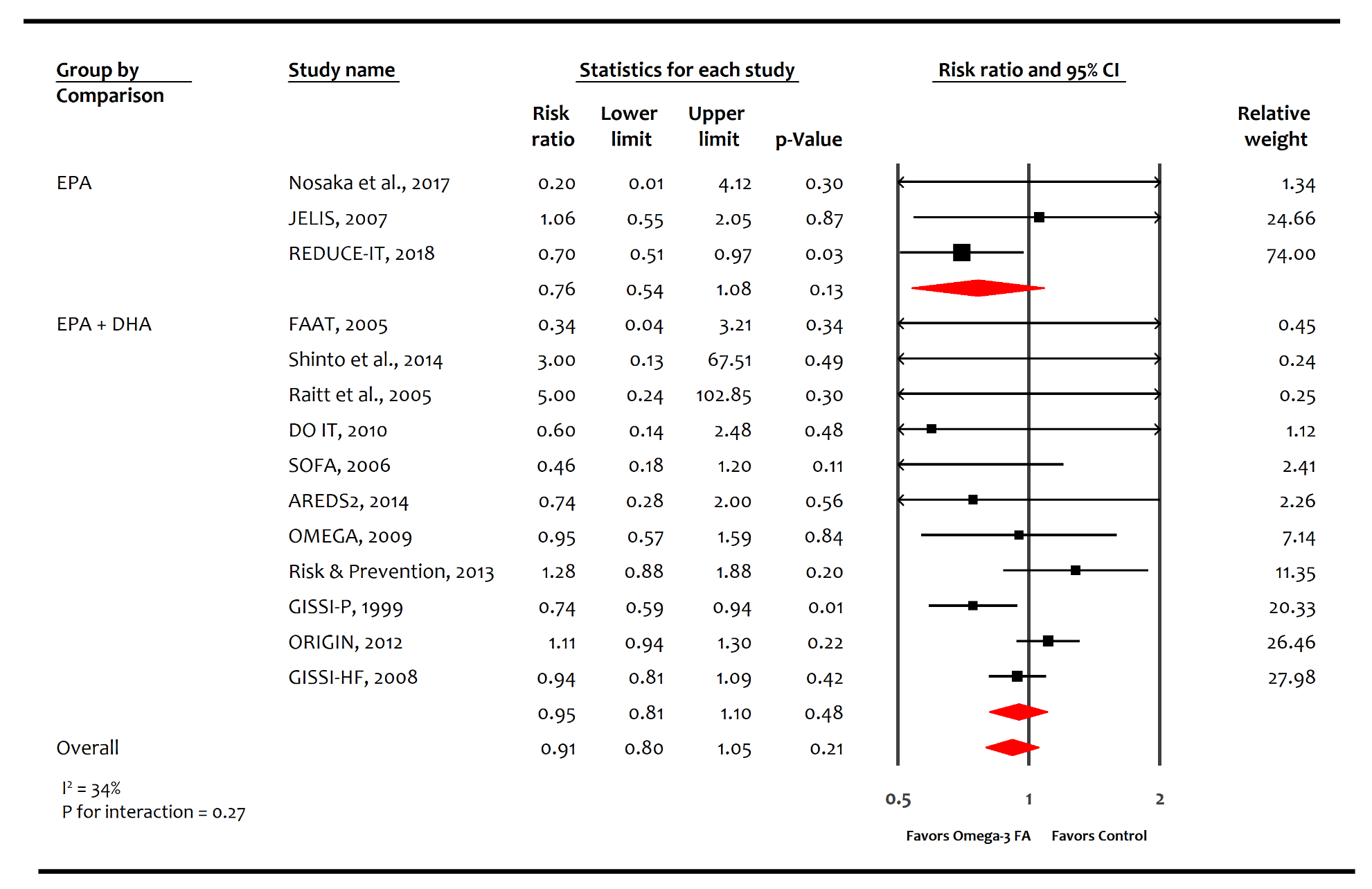


CI: confidence interval; FA: fatty acid. Remaining abbreviations as per webtable 2.


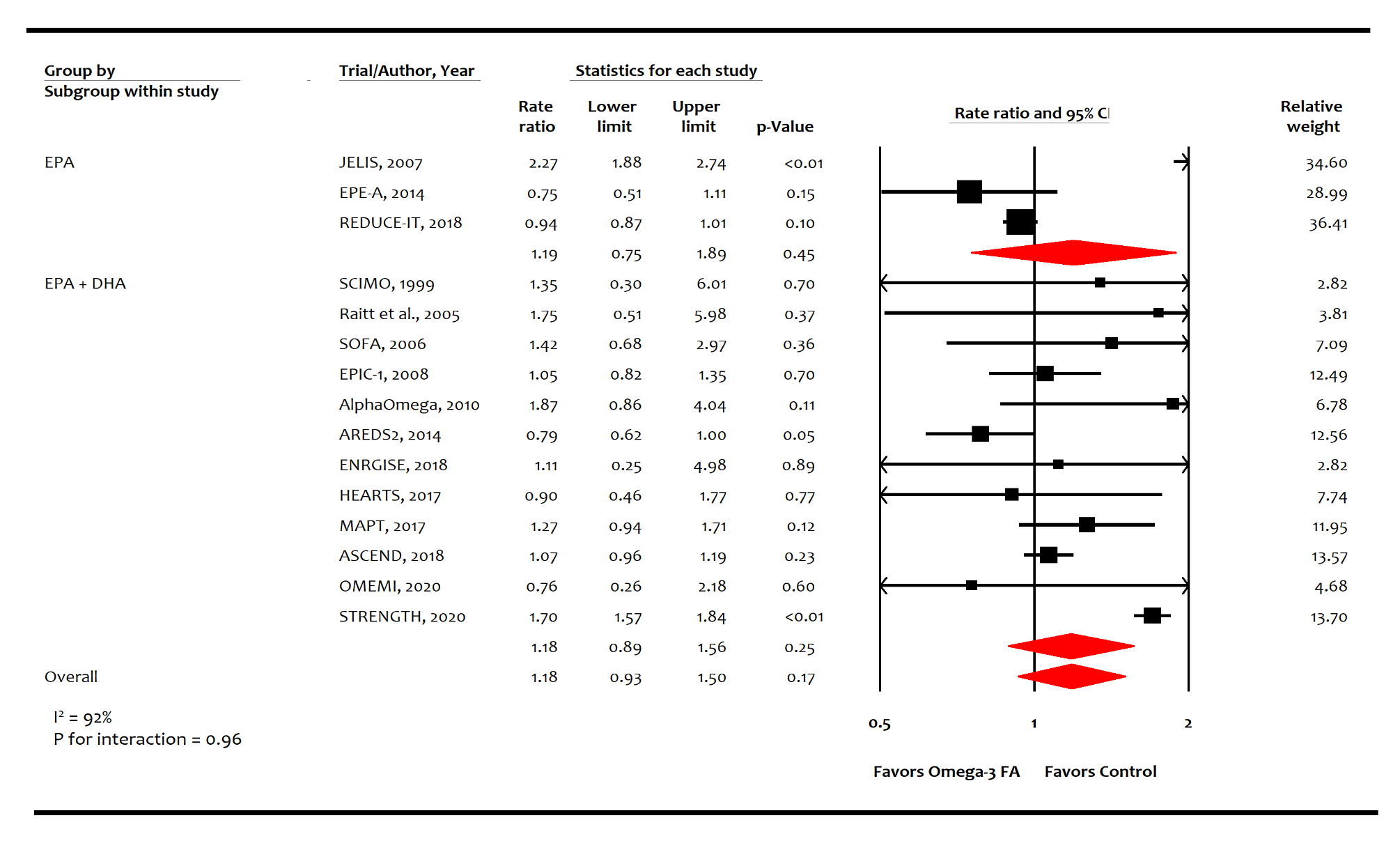
 **Figure 9.** Effect of omega-3 fatty acid on gastrointestinal-related adverse events.

CI: confidence interval; FA: fatty acid. Remaining abbreviations as per webtable 2.


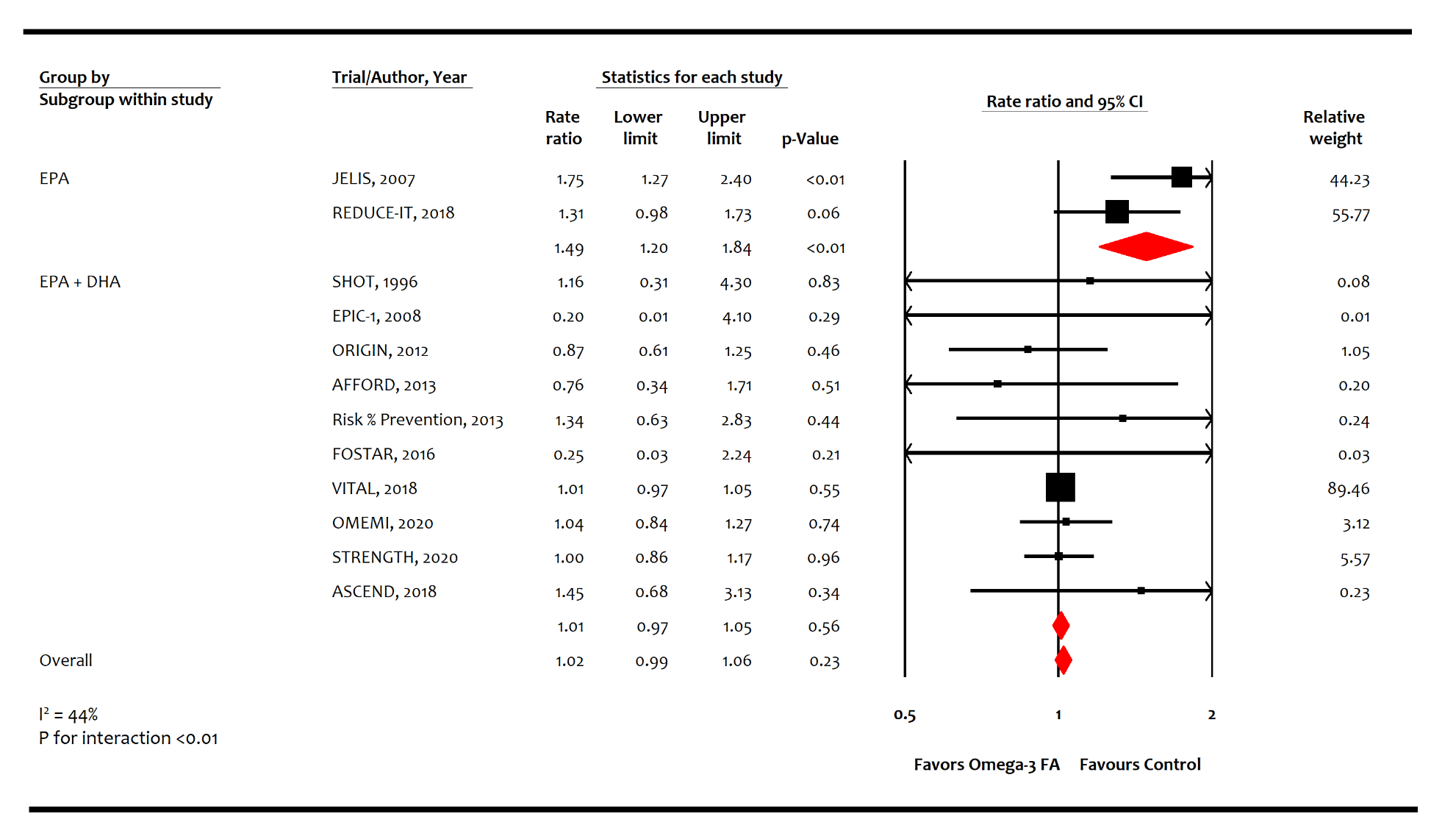
**Figure 10.** Effect of omega-3 fatty acid on total bleeding.

CI: confidence interval; FA: fatty acid. Remaining abbreviations as per webtable 2.


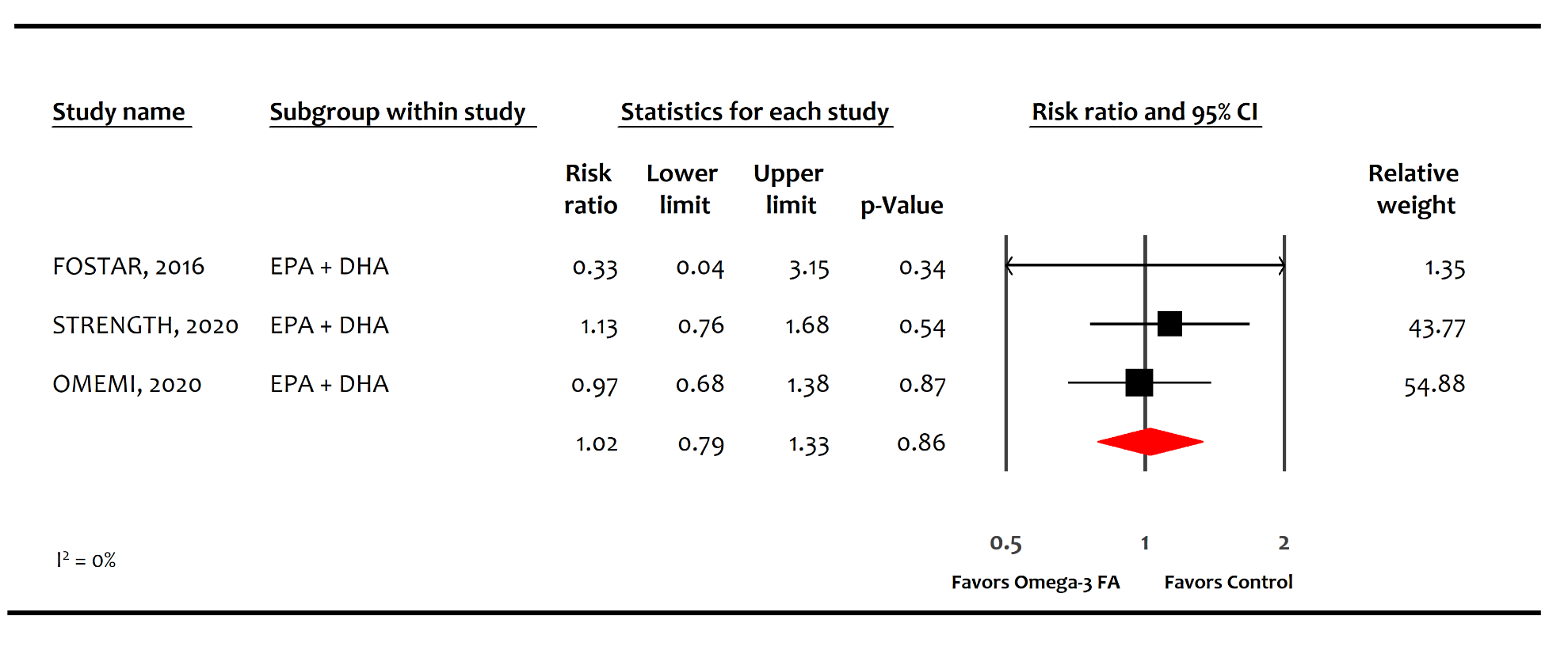
**Figure 11.** Effect of omega-3 fatty acid on major bleeding.

CI: confidence interval; FA: fatty acid. Remaining abbreviations as per webtable 2.


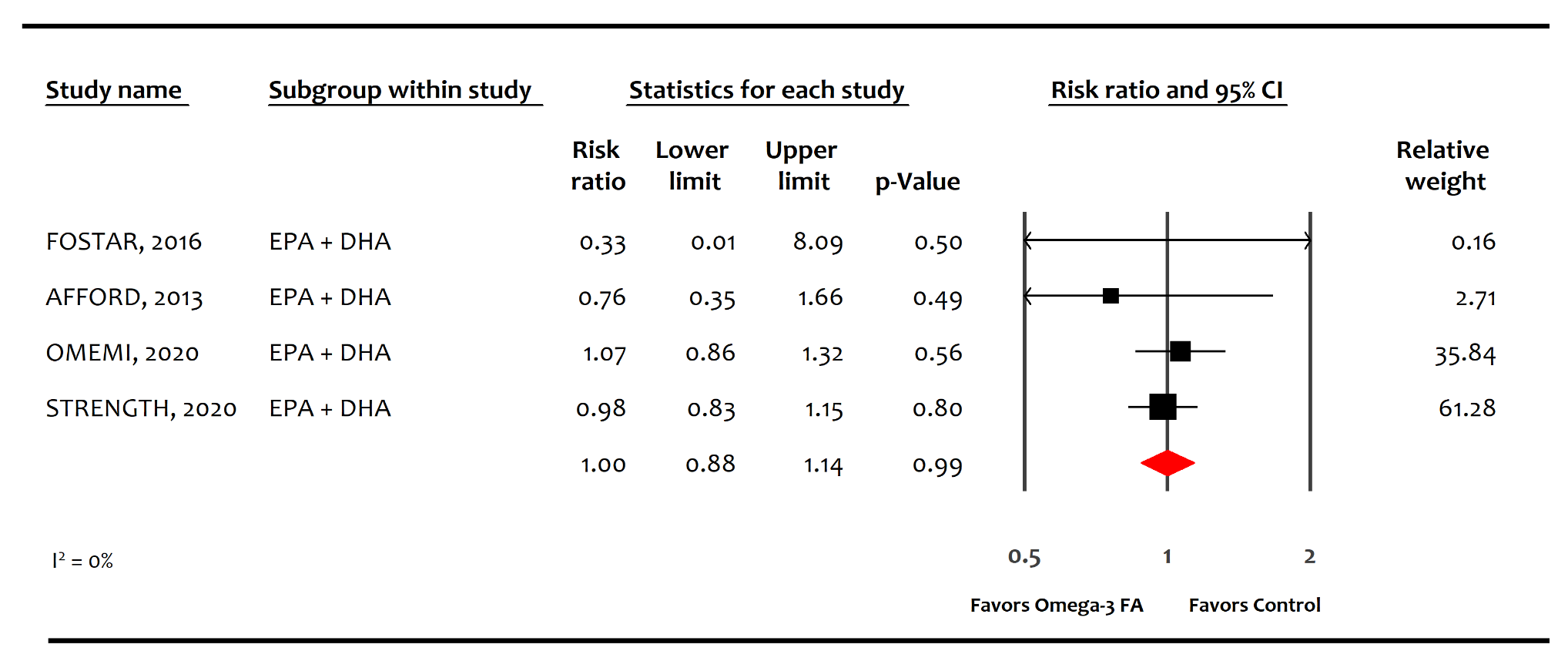
**Figure 12.** Effect of omega-3 fatty acid on minor bleeding.

CI: confidence interval; FA: fatty acid. Remaining abbreviations as per webtable 2.

**Figure 13.** Funnel plot assessing publication bias for non-fatal myocardial infarction.


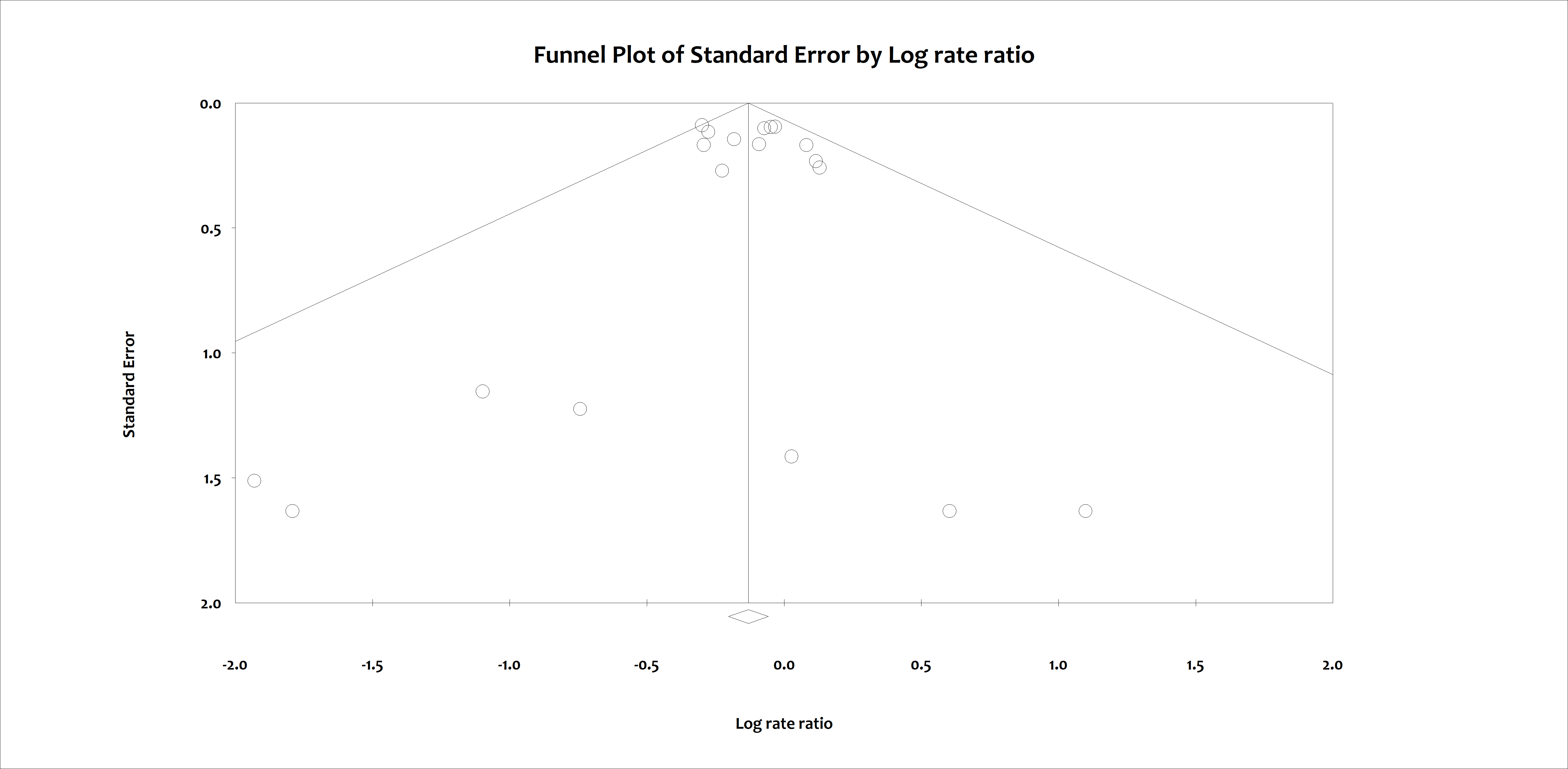


Egger’s P- value (2-tailed): 0.92

**Figure 14.** Funnel plot assessing publication bias for non-fatal coronary heart disease events.


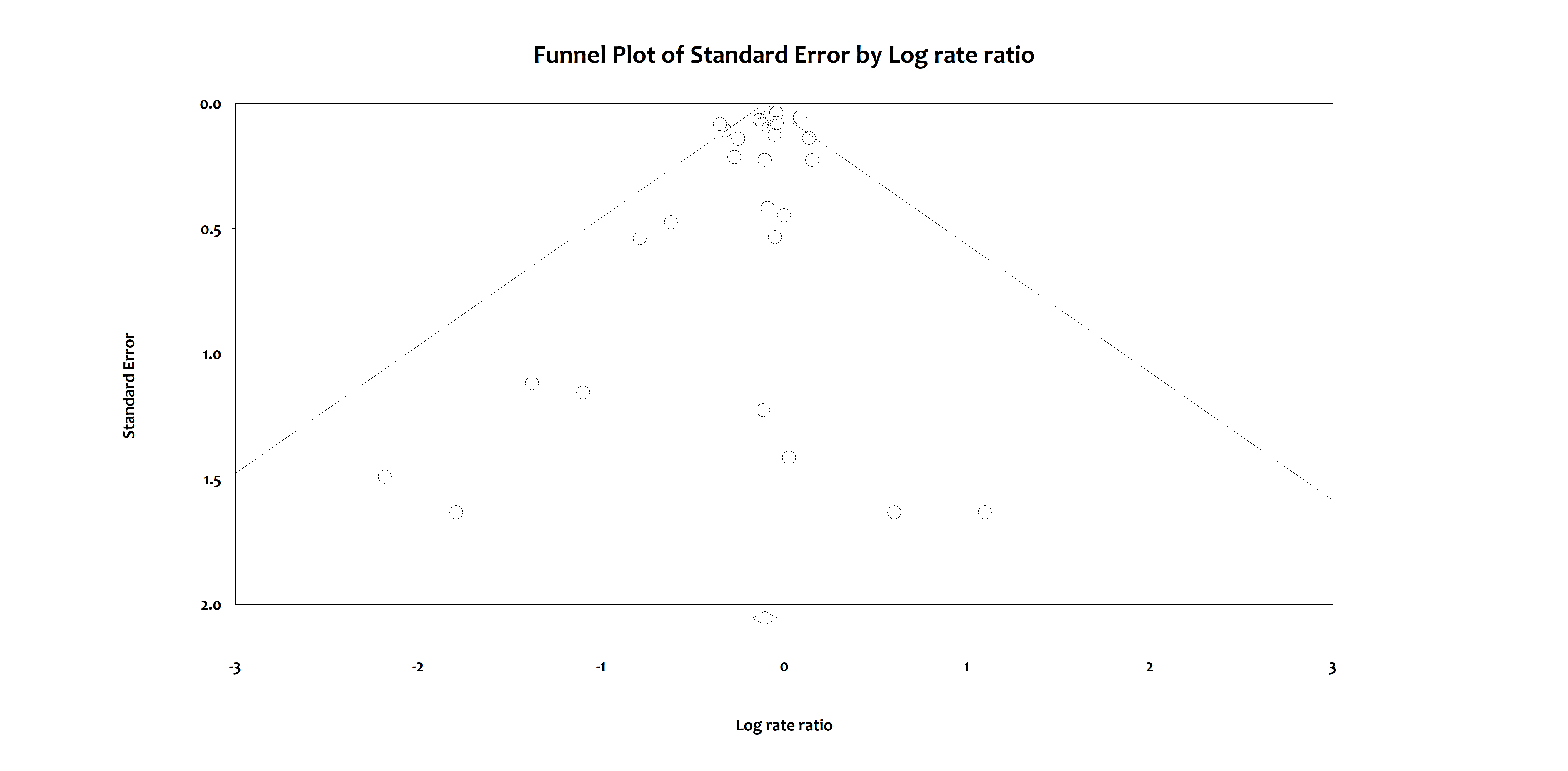


Egger’s P- value (2-tailed): 0.12

**Figure 15.** Funnel plot assessing publication bias for cardiovascular mortality.


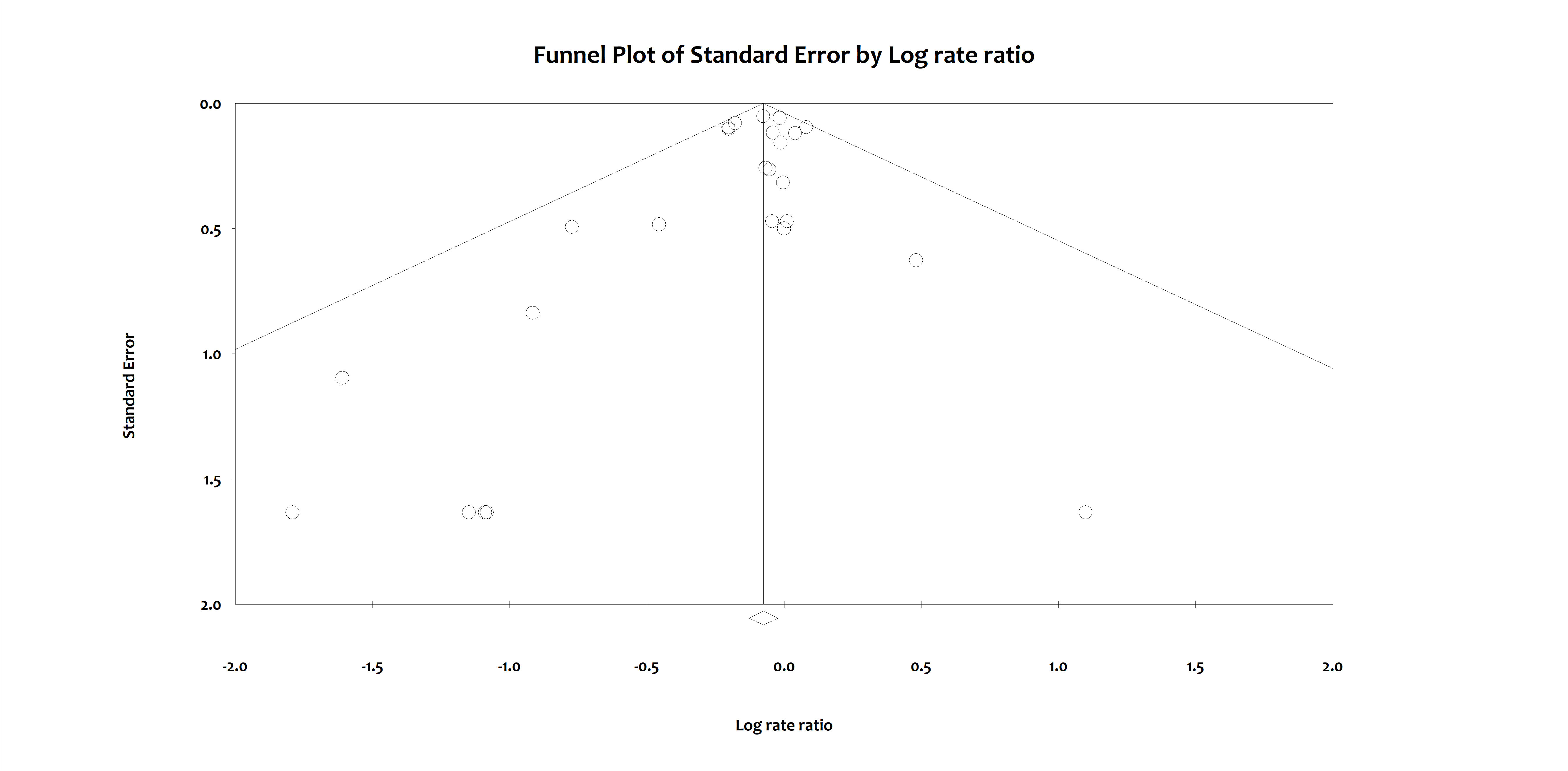


Egger’s P- value (2-tailed): 0.26

**Figure 16.** Funnel plot assessing publication bias for all-cause mortality.


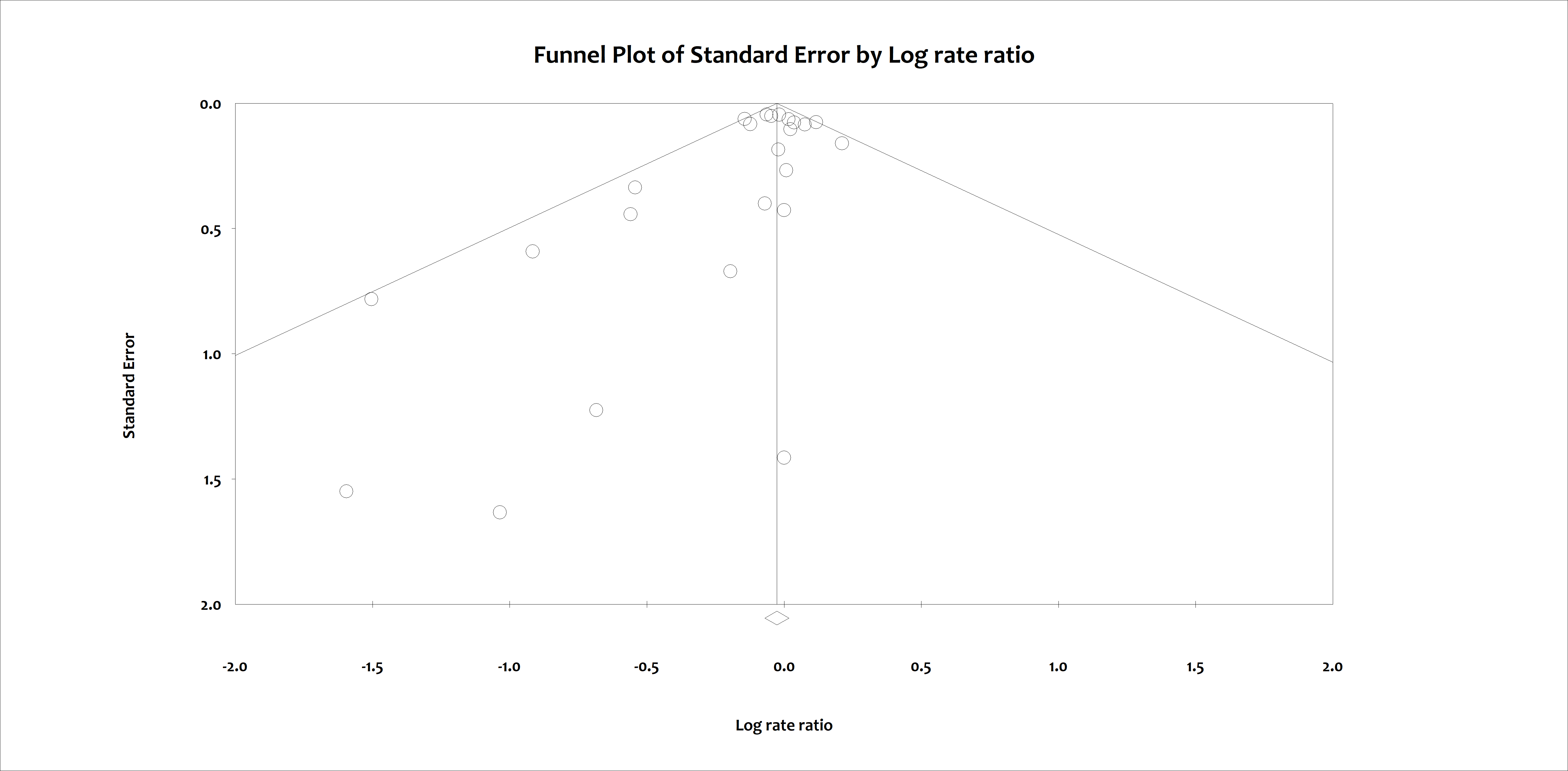


Egger’s P- value (2-tailed): 0.11

**Figure 17.** Funnel plot assessing publication bias for major adverse cardiovascular events (MACE).


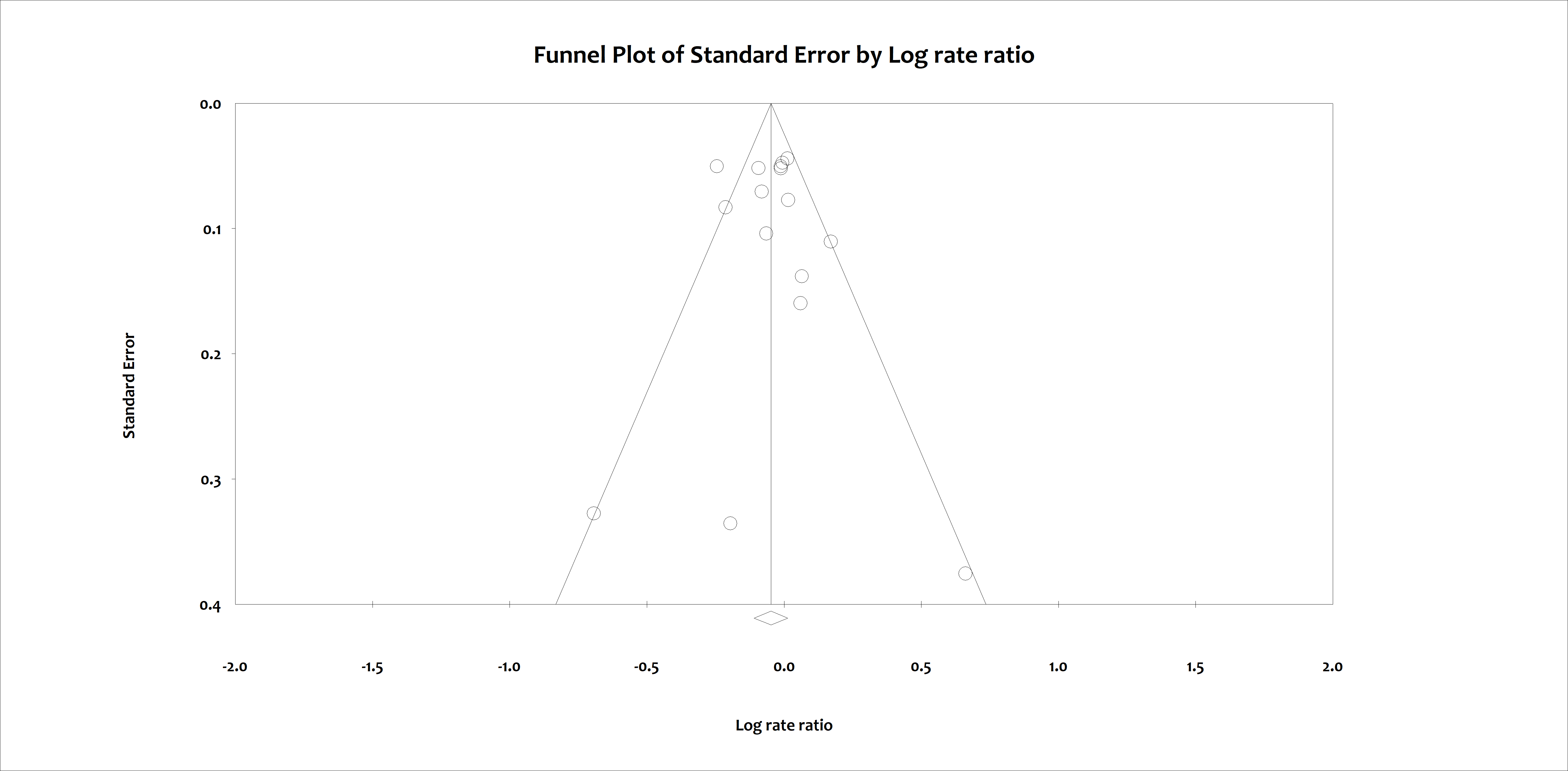


Egger’s P- value (2-tailed): 0.87

**Figure 18.** Funnel plot assessing publication bias for revascularization.


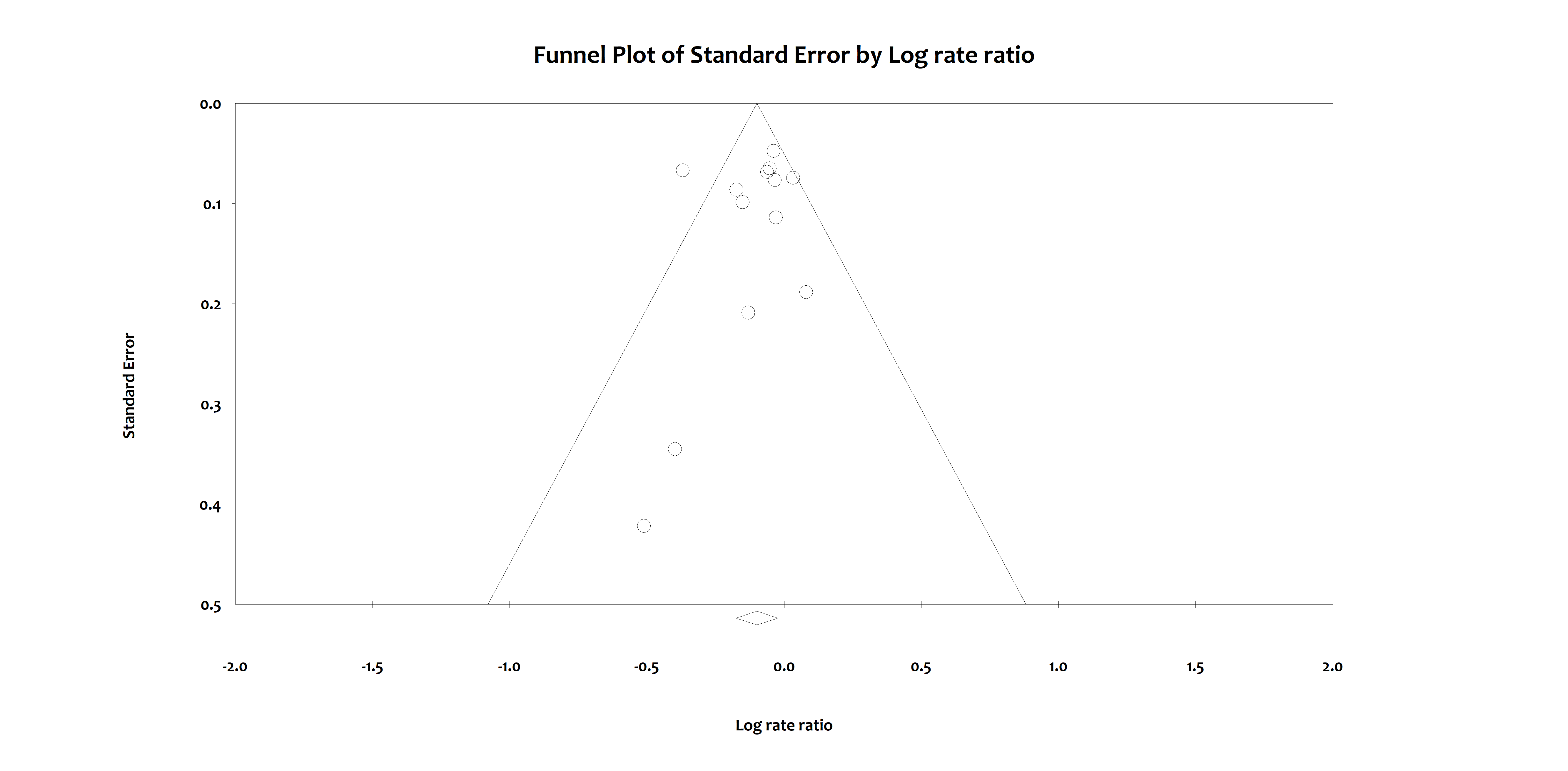


Egger’s P- value (2-tailed): 0.57

**References**

1. Abdelhamid AS, Brown TJ, Brainard JS, et al. Omega-3 fatty acids for the primary and secondary prevention of cardiovascular disease. *The Cochrane Database of Systematic Reviews* 2020; **3**(2): Cd003177.

2. Guyatt GH, Oxman AD, Kunz R, et al. GRADE guidelines 6. Rating the quality of evidence--imprecision. *Journal of Clinical Epidemiology* 2011; **64**(12): 1283-93.

3. Abdelhamid AS, Brown TJ, Brainard JS, et al. Omega-3 fatty acids for the primary and secondary prevention of cardiovascular disease. *The Cochrane Database of Systematic Reviews* 2018; **7**(7): Cd003177.

4. Bernasconi AA, Wiest MM, Lavie CJ, Milani RV, Laukkanen JA. Effect of Omega-3 Dosage on Cardiovascular Outcomes: An Updated Meta-Analysis and Meta-Regression of Interventional Trials. *Mayo Clinic Proceedings* 2020.

5. Koskinas KC, Siontis GCM, Piccolo R, et al. Effect of statins and non-statin LDL-lowering medications on cardiovascular outcomes in secondary prevention: a meta-analysis of randomized trials. *European Heart Journal* 2017; **39**(14): 1172-80.
